# Which glaucoma patients should be monitored at home and exploration of clinician perceptions on home monitoring? a survey of glaucoma specialists in the UK

**DOI:** 10.1136/bmjopen-2023-080873

**Published:** 2024-11-18

**Authors:** Uma Alagappan, Carrie Stewart, Augusto Azuara-Blanco, Anthony J King, Andrew Tatham, Rodolfo Hernández, Graeme MacLennan, Darian Shotton, Mark Forrest, Katie Gillies

**Affiliations:** 1 Aberdeen Centre for Evaluation, University of Aberdeen, Aberdeen, UK; 2 Centre for Public Health, Queen's University Belfast, Belfast, UK; 3 Department of Ophthalmology, Nottingham University Hospitals NHS Trust, Nottingham, UK; 4 Ophthalmology, NHS Lothian, Edinburgh, UK; 5 Health Economics Research Unit (HERU), University of Aberdeen Health Services Research Unit, Aberdeen, UK

**Keywords:** Patients, Glaucoma, Medical ophthalmology, Surveys and Questionnaires

## Abstract

**Abstract:**

**Objectives:**

To identify suitable patients for glaucoma home monitoring and explore clinicians’ perceptions of the possible benefits and risks of home monitoring within the National Health Service.

**Design:**

An online survey composed of open-ended and closed-ended questions.

**Setting:**

Secondary care.

**Participants:**

Glaucoma specialists registered with the UK and Eire Glaucoma Society.

**Outcome measures:**

Agreement with clinical scenarios.

**Results:**

The estimated response rate was 68% (n=49). Of 49 participants, 92% (n=45) were consultant ophthalmologists and 71% (n=35) had over 10-year experience. There was a poor agreement regarding an ideal glaucoma patient for home monitoring, with only one scenario achieving over 60% agreement. Most participants believed that home monitoring would be most suitable for low-risk scenarios, rather than high-risk, due to fear of missing progression. In relation to acceptability, key facilitators included the potential to increase healthcare capacity and promote patient safety. However, low clinician trust in equipment reliability and fear of patient harm were reported as concerns.

**Conclusions:**

There was no clear consensus on which patients would benefit most from glaucoma home monitoring. While many clinicians believe home monitoring may enhance healthcare, there were also many concerns about the technologies themselves. Further work to address clinician concerns is warranted.

**Research Registry registration number:**

6213.

**STRENGTHS AND LIMITATIONS OF THIS STUDY**

- This study exclusively recruited glaucoma specialists, with the most relevant knowledge and expertise in this field, to address our proposed research aims.
- The survey design provided data and understanding about whether certain glaucoma patients should be monitored at home using digital technology and why.
- This study was limited by evidence of survey fatigue, demonstrated by fluctuations in participant engagement throughout the responses.

## Introduction

 Glaucoma is the leading cause of irreversible blindness worldwide and the second most common cause of blindness in the UK.^1^ Glaucoma is an intraocular pressure (IOP)-related optic neuropathy, leading to damage of the optic nerve and peripheral visual field (VF) loss.^1^ Glaucoma commonly affects older adults and is increasing in prevalence in line with an ageing population.^2^ In current practice, the two main measurements used in glaucoma assessment are IOP and VF testing. Patients typically require lifelong monitoring and are usually requested to attend monitoring every 6 months.^2^ The National Health Service (NHS) cannot meet the increasing demand for glaucoma services in their current format.^3^ Finding practical alternatives to in-clinic monitoring is imperative for efficient patient care. One possible solution to decrease demand on glaucoma monitoring services is through making use of digital home monitoring technologies.

Recent technological advances are increasing the possibilities of using patient-led home monitoring, with the 10-year NHS plan identifying chronic condition home monitoring as a priority area.^4^ Several qualitative studies have reported successes for chronic conditions such as diabetes and hypertension.^5^,^8^ Within ophthalmology, the exploration of digital innovation for home monitoring is gathering pace. The Eyecatcher study^5^ reported that home-based VF monitoring improved patient compliance and convenience. However, despite the optimism surrounding the potential for ophthalmological home monitoring, present evidence is insufficient to support initiation of home monitoring into current practice.^8^

Previous studies^5^ have explored patients’ perspectives of glaucoma home monitoring but scarce research into clinicians’ perceptions is available. Clinicians are important stakeholders who can provide insights as to how an intervention can work and raise potential risks. Their support of new interventions will influence the likelihood of success if implemented.

Evidence suggests that the demand for glaucoma services will continue to expand, with longer monitoring periods predicted in the future.^3^ This has led to delays in follow-up appointments, ultimately resulting in evidence showing irreversible visual loss which could have been prevented with adequate monitoring.^3^ There is limited guidance in the literature as to which patients would be the ideal for home monitoring. Identifying uncertainties regarding patient suitability is a critical first step towards evaluating its use.

This manuscript reports a study that is part of the National Institute for Health and Care Research (NIHR) Health Technology Assessment funded multiphase In-home Tracking of glaucoma: Reliability, Acceptability and Cost study.^9^ The larger In-home Tracking of glaucoma: Reliability, Acceptability, and Cost (the I-TRAC Study) aimed to identify whether the use of digital technology to home monitor glaucoma is feasible and acceptable to a range of relevant parties through a mixed-method study. The project involved the use of two home monitoring technologies, the iCare HOME, a handheld tonometer to measure IOP and the OKKO Health App to measure VFs.

The first objective of the ITRAC study,^9^ the results of which are reported within this manuscript, was through a glaucoma specialist survey which aimed to identify suitable patients for glaucoma home monitoring and explore clinicians’ perceptions of the possible benefits and risks of home monitoring.

## Methods

### Data collection

#### Participants

Eligible participants were glaucoma specialists (ophthalmologists and optometrists) as they are directly involved in the monitoring and management of patients, therefore, allowing clinically relevant opinions to be evidenced.

We believe that patients are additional key stakeholders within this topic, however, we have explored their perspective within the larger work of the ITRAC monograph.^9^

#### Recruitment

The survey was disseminated via UK and Eire Glaucoma Society (UKEGS), a non-profit professional society for clinicians with a specialist interest in glaucoma. A survey link was emailed to UKEGS members by the UKEGS Communications Manager. In addition to the UKEGS email, our clinical principal investigators (PIs) raised awareness of the questionnaire among clinical networks and the study was promoted via social media. The survey was active from 14 May 2021 to 30 October 2021.

Based on the estimated number of clinicians registered with UKEGS (range n=69–72),^10 11^ we are accepting our denominator for calculating the estimated response rate as n=72. UKEGS does not currently record the designation of its members so the exact number of specialists surveyed is unknown.

#### Design

The online questionnaire used both open-ended and closed-ended questions. The initial data collection used a combination of closed-ended vignettes, to quantitatively investigate which patient’s clinicians would deem suitable for home monitoring, and open-ended free-text questions to assess overall perspectives. Following this, separated data analysis permitted generation of quantitative frequencies and percentages in relation to agreement among clinicians regarding patient selection while also qualitatively assessing free-text responses regarding home monitoring for theme creation. Findings were then integrated at the interpretation phase by justifying quantitative findings with qualitative responses.

#### Device selection

The two technologies initially selected to be explored within this study were the iCare HOME tonometer and the MRF app, accessed via an iPad to measure VFs. However, the MRF app was not CE marked so instead replaced with the OKKO health app.

We predicted that the OKKO health app would have transferable findings to the MRF app, considering it is also an app-based VFs device; however, understand that the lack of assessment was not ideal.

Previous studies assessing the iCare tonometer found that most participants were able to correctly use the device following training^12^,^16^ Additionally, iCare has been compared against the Goldmann automated tonometer (GAT) in numerous studies. Overall, the measurement differences reported between GAT and iCare vary between −2.7 and 0.7 mm Hg.^1214^,^18^ Importantly, variations of 0–5 mm Hg are considered acceptable ranges for home monitoring.^13^,^18^

#### Questionnaire

An online survey (see online supplemental Information) was created through Survey Monkey.^19^ Information on ITRAC and home-monitoring devices was included at the start of the survey to ensure participants were given contextual insights to promote informed responses. Additionally, a summary of the National Institute for Health and Care Excellence guidelines for ocular hypertension and primary open-angle glaucoma, along with an introduction to the technologies being discussed (iCare Home Tonometer and the OKKO tablet-based app for measuring VFs), was included.^2^ Peer-reviewed evidence for the iCare tonometer was referenced within the leaflet^12^,^18^ but there is yet to be published evidence regarding the OKKO app. Demographic data collected included age, gender, ethnicity, clinical profession type and the number of years’ experience in treating glaucoma.

Clinicians were asked to decide if they would use the iCare home tonometer and/or the OKKO App for VFs, for each of four clinical scenarios (designed to represent varying disease severity). Additionally, clinicians were asked to report suggested frequency and total duration of home monitoring for each. The patient scenarios (described in table 1) included patient demographic information and hypothetical details on glaucoma severity (mild, moderate and severe), current treatment, disease control (apparently well controlled, uncertain, poor) and management options. These cases were developed by three clinical authors (AA-B, AJK and AT) and designed to reflect NHS guidelines for glaucoma care.^10^ In addition, clinicians were asked to explain the rationale behind rejecting home monitoring for each. The order of scenarios presented within the survey was randomised.

**Table 1:** A description of the four clinical vignettes presented in the survey

	Scenario 1	Scenario 2	Scenario 3	Scenario 4
Name	Mr Smith	Ms Adams	Mr Patel	Ms McEwen
Age	63	70	78	55
Gender	Male	Female	Male	Female
Brief history	2-year history of severe bilateral glaucoma. No evidence of current progression	1-year history of bilateral ocular hypertension	3-year history of poorly controlled pseudoexfoloiation in R eye and moderate glaucoma in L eye	5-year history of mild, bilateral normal tension glaucoma. No progression noted
Intended level of glaucoma progression risk	High risk	Low risk	High risk	Low risk

**Table 1:** Please note that the intended level of glaucoma progression risk was not provided to survey participants.

A fifth scenario was presented with a hypothetical model involving home monitoring to explore acceptability more broadly, without involving specificities of hypothetical patients as with the previous vignettes. It described a clinician who does not have sufficient clinic capacity, and therefore, cannot monitor his patients at recommended intervals. Due to this, he decided to concentrate on patients with disease progression in the last 2 years and those with uncontrolled IOP. The remaining patients with stable disease would use home monitoring with the proposed devices. He would then review these results every 6–12 months in ‘virtual clinics’. Survey participants were asked whether they felt this hypothetical model of care was acceptable.

Scenario 5 was proposed with the aim of identifying perceived barriers and facilitators of home monitoring (allowing creation of themes through the free-text responses), whereas the clinical vignettes were used in attempt of researching whether there was agreement among clinicians regarding which patients could be suitable for home monitoring. If scenarios were left completely open ended, the responses may have been too wide to collate any valuable themes for analysis.

#### Consent

Participants were asked to provide consent at the start of the questionnaire. Failure to provide this prevented progression into the questionnaire.

#### Data management

Data were downloaded and stored in an Excel worksheet. Participants were assigned a unique identifier number on their questionnaire.

#### Patient and public involvement

The ITRAC study has a patient collaborator (DS) as part of the study team, DS was a coapplicant for the NIHR funding and was involved in study development and design. The project has further patient and public involvement representation in our study steering group from Glaucoma UK and one further patient partner. DS has been involved in study planning prior to funding award and helped inform the development of study design.

#### Analysis

Quantitative data were analysed using descriptive statistics (eg, frequencies, percentages). Agreement within clinical scenarios was defined by the study team as being ≥60% in supporting or not supporting the hypothetical patient to be home monitored. We chose this value based on team discussion and on best judgement that over half of the respondents agreed.

The six-phase Braun and Clarke approach to thematic analysis was adopted to analyse free-text responses.^20^ We used inductive and deductive coding to build themes. For inductive, codes are developed based on searching for similar issues within data. For deductive codes, we looked for existing concepts based on previous work. For both, we reviewed the data from the perspective of identifying barriers and facilitators. Barriers were defined as features of the intervention itself or the environment it would be implemented within which can or has the potential to prevent or limit the utility of the intervention. Facilitators were defined as features of the intervention itself or the environment it would be implemented within which can or has the potential to permit or enhance the utility of the intervention.

UA and CS reviewed the free-text data, noting the points being made in responses as codes, which were reviewed for similarities and differences. UA and CS then developed a list of themes, groups of interconnected codes, for barriers and facilitators. Once provisional themes were developed, UA designed a coding framework to define themes. Themes are generally descriptive rather than analytical, reflecting the limited qualitative data available and the inability to check understanding or explore points raised further. This was intentional so as to link the findings from this work to subsequent phases of ITRAC and to use these descriptive themes as areas for further exploration in the interviews and focus groups. Descriptive themes often incorporate barriers and facilitators reflecting divergence in participant views.

The development of themes was supported by the use of QSR NVivo program.^21^ The coding framework, listing the themes their codes and their descriptions, was updated throughout the analytical process. For rigour, themes were reviewed and agreed by the team.

## Results

A total of 64 clinicians responded to the survey. Three participants were excluded based on lack of meeting inclusion criteria. For example, glaucoma nurses were excluded as they did not directly make clinical decisions regarding monitoring or management for glaucoma patients, despite having an interest in glaucoma. Another participant did not respond to the screening question and was additionally excluded. A further 11 participants were excluded from the final analysis as they did not respond to the clinical scenario questions and only provided demographic data. Therefore, 49 clinicians who replied to at least one of the questions in relation to the clinical scenarios were included in the final analysis. As shown in table 2, most participants were white (59%, n=29), male (69%, n=34), consultants (92%, n=45), aged between 50 and 59 (45%, n=22), who have treated glaucoma patients for >10 years (71%, n=35).

**Table 2:** Participant demographic summary table (n=49)

Variables	Number of responses, n (%)
Total no of participants	49 (100.0)
Duration of experience with treating glaucoma	
<5 years	3 (6.1)
5–10 years	11 (22.4)
>10 years	35 (71.4)
Profession	
Optometrist (glaucoma specialist)	4 (8.2)
Consultant ophthalmologist	45 (91.8)
Participant age	
<40	7 (14.3)
40–49	16 (32.7)
50–59	22 (44.8)
>60	4 (8.2)
Gender identity	
Male	34 (69.3)
Female	13 (26.5)
Non-binary	1 (2.0)
Prefer not to say	1 (2.0)
Ethnicity	
White	29 (59.2)
Mixed white and black African	3 (6.1)
Asian/Asian British	16 (32.7)
Black/black British African	1 (2.0)

### Can a target group of patients who would be most suited for glaucoma home monitoring can be defined?

When comparing the support for and against home monitoring for each of the four clinical vignettes (online supplemental figure 1) agreement among clinicians (within scenarios) could not be determined. Only scenario 4 met our definition of agreement (≥60%). This scenario hypothetically presented a stable, low-risk patient (Mrs McEwen) with normal tension glaucoma (NTG). She had a mild disease and had not progressed in 5 years. In this scenario, 61% (n=30) of participants would refer her to use the iCare tonometer and 65% (n=32) would promote the use of the OKKO app.

Participants were asked to suggest optimal time frames for the frequency and duration of home monitoring (of both IOP and VF) through open-ended questions in each scenario (see table 3). A wide spectrum of durations was suggested for the low-risk scenarios (1 and 3), shorter frequency time frames were suggested for home monitoring at every 1–7 days with a duration of 2–6 months. The high-risk scenarios (2 and 4) mainly had longer frequencies of monitoring, at every 2–6 months with a duration of around 1 year.

**Table 3:** Clinician decisions regarding patient suitability for glaucoma home monitoring

	Scenario 1Mr. SmithHigh risk(n)	Scenario 2Ms. AdamsLow risk(n)	Scenario 3Mr. PatelHigh risk(n)	Scenario 4Ms. McEwanLow risk(n)
Would you consider it useful to monitor IOP at home?				
Yes	26 (53%)	28 (57%)	25 (52%)	30 (61%)
No	23 (47%)	18 (36%)	18 (36%)	14 (9%)
No response	0 (0%)	3 (6%)	6 (12%)	5 (10%)
Recommended frequency of IOP monitoring				
Every 1–7 days	19 (73%)	9 (32%)	13 (50%)	7 (23%)
Monthly	3 (12%)	3 (11%)	3 (12%)	4 (13%)
Every 2–6 months	3 (12%)	12 (43%)	7 (27%)	13 (43%)
Every 7–12 months	0 (0%)	0 (0%)	0 (0%)	4 (13%)
Unclear	1 (5%)	3 (11%)	3 (12%)	2 (6%)
No response	0 (0%)	1 (3%)	0 (0%)	0 (0%)
Recommended duration of IOP monitoring				
Daily	2 (8%)	1 (3%)	2 (8%)	1 (3%)
Weekly	4 (15%)	1 (3%)	3 (12%)	2 (6%)
Monthly	4 (15%)	0 (0%)	3 (12%)	1 (3%)
Every 2–6 months	8 (30%)	3 (11%)	10 (38%)	4 (13%)
Every 7–24 months	1 (5%)	13 (46%)	5 (19%)	17 (57%)
Unclear	7 (27%)	7 (25%)	2 (8%)	3 (10%)
No response	0 (0%)	3 (11%)	0 (0%)	2 (6%)
Would you consider it useful to monitor visual function at home?				
Yes	28 (57%)	26 (53%)	20 (41%)	32 (65%)
No	19 (39%)	18 (37%)	24 (49%)	12 (24%)
No response	2 (4%)	5 (10%)	5 (10%)	5 (10%)
Recommended frequency of visual function monitoring				
Every 1–7 days	4 (14%)	0 (0%)	2 (10%)	1 (3%)
Monthly	11 (39%)	5 (19%)	4 (20%)	5 (16%)
Every 2–6 months	8 (29%)	14 (54%)	10 (50%)	20 (63%)
Every 7–12 months	0 (0%)	4 (15%)	1 (5%)	3 (9%)
Unclear	5 (18%)	3 (12%)	2 (10%)	2 (6%)
No response	0 (0%)	0 (0%)	1 (5%)	1 (3%)
Recommended duration of visual function monitoring				
Daily	0 (0%)	0 (0%)	1 (5%)	0 (0%)
Monthly	2 (7%)	0 (0%)	0 (0%)	0 (0%)
Every 2–6 months	17 (61%)	4 (15%)	10 (50%)	4 (13%)
Every 7–24 months	2 (7%)	16 (62%)	3 (15%)	22 (69%)
Unclear	3 (11%)	2 (8%)	3 (15%)	5 (16%)
No response	4 (14%)	4 (15%)	3 (15%)	1 (3%)

Please note that the frequency refers to how often monitoring would be performed within the total duration of monitoring (ie, twice a week for a total of 6 months).

IOPintraocular pressure

The response rates at varying points of the survey were monitored to identify any signs of survey fatigue. The initial demographics and scenario 1 response rates were at 100% (n=49). However, by scenario 3, the rate dropped to 88% (n=43). Despite this, scenario 4 had a response rate of 90% (n=44), indicating fluctuations in engagement.

To explore if the disagreement in scenarios was related to the digital technology itself or patient selection, we compared agreement across scenarios. There was a lack of consensus relating to which patients should be monitored using iCare, with 23 (47%) reporting that home monitoring would be useful in at least three of the four scenarios. This was also true for VFs with 22 (45%) of clinicians believing it to be useful for three out of the four scenarios.

We asked participants to describe their reasoning if they deemed a scenario unsuitable (table 4). The participants who expected Mrs McEwen, scenario 4, to be suitable for home monitoring justified their views due to the nature of her stable disease and her low risk of complications (such as disease progression). They felt that she could be safely monitored at home without a high risk of missed progression, which could allow increased clinic capacity for patients with unstable IOPs or progressing disease.

**Table 4:** Summary of participant justification responses for deeming each patient scenario unsuitable for home monitoring

Scenario	Participant justification	Example quote
1Mr. Smith high risk	Advanced/high-risk glaucoma Requires treatment, not monitoring Risk of increased patient anxiety Requires full comprehensive in-person assessment	*‘*Severe glaucoma in a relatively young patient on maximum medical treatment; IOP control borderline although VF is stable it is for a relatively short duration. Need more fields to establish long-term stability candidate for HES (hospital eye services) care*’* Participant: Consultant, with 5–10 years of experience
2Ms. AdamsLow risk	Stable, OHT patient Low risk of conversion to glaucoma Most would discharge to community care	*‘*In our unit this pt would be discharged to community optometry glaucoma service and would recommend optom to see 24 monthly.’ Participant: Consultant with >10 years of experience
3Mr. PatelHigh risk	Advanced/high-risk glaucoma More likely to struggle with home monitoring due to frailty/compliance Requires treatment and assessment rather than monitoring Risk of increased patient anxiety	If his visual fields are not reliable in the clinic, then we would have to test out if he was any better with the home version before considering. If not, then I would leave it and do OCT in the clinic. Participant: Consultant with >10 years of experience
4Ms. McEwanLow risk	NTG, stable (5 years) patient Low risk of progression May be a waste of resources	‘Low risk—not worth the extra resources’ Participant: Consultant with 5–10 years of experience

NTG, normal tension glaucomaOHT, ocular hypertensionVFvisual field

Freeing up capacity in the hospital eye service, allowing better use of resources and enabling better care of high-risk patients. Low-risk patients may prefer not having to come in the hospital.

Participant: Consultant with 5–10 years of experience

Among the participants who reported that this patient scenario would be unsuitable for home monitoring, a common justification was that it may be a waste of resources due to her stable condition. This was a contradiction with other participants who deemed her suitable for the same reasoning.

Poor resource use, waste of effort, low risk—not worth the extra resources

Participant: Consultant with 5–10 years of experience

When evaluating the rationale behind participants deeming other scenarios unsuitable, the main concerns contradicted each other (see table 4)—much like in the case of scenario 4. However, many (n=32) highlighted that they may be more hesitant to recommend home monitoring to high-risk patients. They predicted that they would rather assess these patients in the clinic to confidently determine their measurements and avoid any discrepancies due to potentially ‘unreliable’ readings. They also felt that these advanced cases may require additional resources, such as imaging.

High risk of further vision loss within lifetime—young, advanced VF defects bilaterally. Would prefer to see in clinic and discuss surgery at each visit—regarding Scenario 1

Participant: Optometrist with >10 years of experience

### The acceptability of glaucoma home monitoring from the perspective of glaucoma specialist clinicians

Scenario 5 described a model combining home monitoring into the current system. Of the 49 participants, 52% (n=26) felt that this model was acceptable. However, the remaining 37% (n=18) felt that it was unsuitable and a further 10% (n=5) did not respond, overall reducing the consensus. Participants were asked to explain their decision through a series of free-text questions around perceived advantages and disadvantages. Thematic content analysis of free-text responses in relation to this scenario resulted in the following seven themes: the impact of home monitoring on resources, the influence of patient characteristics on suitability for home monitoring, the impact of home monitoring tests on clinician confidence in delivering care to patients, clinicians beliefs about how home monitoring may impact patient safety, general perceived facilitators of home monitoring, clinician concern regarding the impact of home monitoring for patients with accessibility barriers and clinician beliefs about the medical suitability of patients for home monitoring. Within each theme, anticipated advantages (facilitators) and disadvantages (barriers) in relation to glaucoma home monitoring were identified (as shown in table 5).

**Table 5:** Perceived barriers and facilitators of glaucoma home monitoring

Theme	Subtheme	Code	B/F	Comments	Participants	Example quote
The impact of home monitoring on resources	Financial cost	Cost-effective	F	2	1	‘In the long-term, it may be cheaper too’
		Poor value	B	28	24	‘Home tonometry for all patients seems a grandiose waste of resources’
	Healthcare capacity	Increased capacity	F	71	36	‘Utilising the limited capacity to see stable patients virtually is helpful to generate more capacity for patients who require more attention’
		Decreased capacity	B	4	4	‘Home monitoring would need to be well supported, to train and supervise patients, and well planned, to review data’
	Inadequate alternatives	–	F	13	11	‘Better to get some monitoring then just being a name on the waiting list and losing sight.’
	Human resources	Staff to train	B	11	11	‘Securing the funding and staffing to train patients and to troubleshoot might be a challenge’
		Staff to review	B	9	9	‘There would be a significant burden in virtually reviewing all these patients which would need to be accounted for in the business case.’
The influence of patient characteristics on suitability for home monitoring	Patient compliance	Increased compliance	F	4	4	‘May empower patient and improve adherence as they get direct feedback on the effects of treatment and status of disease’
		Decreased compliance	B	38	26	‘The governance of non-compliancy with lack of patient involvement would be another challenge’
	Cognitive, physical and mental patient ability	Cognitive ability	B	2	2	‘Forgetting the original treatment instruction’
		Physical ability	B	49	31	‘Patients with reduced mobility/health issues making clinic attendance or VF testing difficult.’
		Decreased anxiety	F	4	3	‘Where they are anxious about something and have phoned in to ask for early review.’
		Increased anxiety	B	4	4	‘They may get very anxious about small changes in results without full understanding.’
The impact of home monitoring tests on clinician confidence in delivering care to patients	Increased confidence	Improved clinician trust in care delivered	F	11	10	‘In reality glaucoma patients may actually do better with more regular IOP and field testing as will pick up discrepancies sooner and we can't to the tests this often in the clinic.’
	Decreased confidence	Reliability issues	B	83	35	‘We do not have enough information about effectivity.’
		Standardised conditions	B	6	5	‘There is a possibility of someone other than the patient performing the home tests and passing it as the patients.’
		Consistency	B	10	9	‘No consistency between hospital and home care tests’
		Limitations of home monitoring	B	34	18	‘OCT not done which may be considered important by some for early disease’
Clinicians beliefs about how home monitoring may impact patient safety	Patient safety profile	Increased clinical safety	F	21	18	‘Greater number of patients getting timely monitoring’
		Fear of patient harm	B	51	29	‘Experience tells us that some patients will lose vision in the virtual system, despite best efforts to risk stratify and see virtually.’
	IT concerns	IT governance risk	B	9	8	‘IT works well when it works well, but more than often there are barriers and incomplete data etc, The governance of non-compliancy with lack of patient involvement would be another challenge’
General percieved facilitators of home monitoring	Environmental benefit	–	F	1	1	‘Good for the planet - low carbon footprint from not having to travel to the hospital.’
	Increased patient convenience	–	F	9	9	‘More convenient for the patient’
	Physical access to clinic	–	F	21	12	‘Bedbound patients in care homes’
Clinician concerns regarding the impact of home monitoring for patients with accessibility barriers	Language	Language barrier	B	17	16	‘Harder to reach patients would still have a low uptake of the technology. Education is more important.’
	Disability	–	B	7	6	‘Also, patients with physical disabilities or learning difficulties/dementia will struggle with home monitoring themselves’
	Remote access	–	B	2	1	‘Internet availability for download of test results.’
Clinicians beliefs about the medical suitability of patients for home monitoring	Stable disease	NTG	F	42	25	‘In established NTG were progression despite good IOP in office measures.’
		OHT	F	10	7	‘Only OHT patients can be managed safely with virtual clinics’
		Screening	F	7	7	‘Also useful as screening test’
		Care change monitoring	F	11	11	‘May be particularly useful immediately after diagnosis or after change in treatment to determine rate of progression’
		Phasing (24-hour monitoring)	F	17	15	‘Patients with progressive glaucoma - with apparently ‘controlled’ IOP’
		Low-risk suitable	F	22	18	‘Low -medium risk patients can be monitored virtually’
		Low-risk unsuitable	B	26	19	‘Due to the limited capacity in hospital glaucoma clinics, we should focus our resources in higher risk patients.’
	Unstable disease	High-risk suitable	F	8	7	‘Concentrating on riskier cases without losing focus on the well-controlled ones’
		High-risk unsuitable	B	56	32	‘FTF slots kept for those with uncontrolled IOP, high risk, post-op’s etc’

The comments column refers to the number of direct references to each of the codes throughout all survey responses. The final column refers to how many participants (out of the total 49) made the comments referred to in the comment’s column. A dash is used within the table if no code was created for the subtheme.

ITInformation TechnologyNTGnormal tension glaucomaOHTocular hypertension

The key anticipated facilitators of home monitoring include the potential for increased healthcare capacity (n=36, 74%), improved patient safety (n=18, 37%), tackling of physical access barriers to attending clinic (n=12, 25%) and the absence of adequate alternatives (n=11, 23%). From a resource perspective, many clinicians believe that implementing home monitoring could increase capacity for high-risk patients and believe that home monitoring could be better than the alternative of no monitoring. Participants believed that home monitoring may allow patients to access monitoring in times of restriction, such as lockdowns. Many clinicians also anticipated that access to timely care via home monitoring, in a system that cannot currently provide, could prevent irreversible blindness with regular monitoring—overall promoting patient well-being.

The key anticipated barriers participants identified included potential reliability concerns (n=35, 71%)), patients being physically unable to undertake home monitoring (n=31, 63%), fear of patient harm (n=29, 59%) and decreased patient compliance (n=26, 53%). Our findings suggest that clinicians were worried about how home monitoring may impact on quality of care, discussed through concerns about device/data reliability, standardisation and compatibility, and the potential for missed disease progression. They also had concerns about patients’ ability to physically use these devices. This was mainly surrounding frailty and dexterity issues in patients of the typical glaucoma demographic, resulting in poor compliance rates.

## Discussion

### Principal findings

To the best of our knowledge, this is the first study to explore clinician perspectives towards glaucoma home monitoring within the UK. The study identified a lack of agreement among participants regarding an ideal patient population for home monitoring. The strongest consensus was seen for a stable, low-risk patient, with >60% of the participants supporting IOP and VF home monitoring. Participants reported that they may not offer home monitoring to high-risk patients, due to the fear of missing disease progression from unreliable readings. However, they predicted that home monitoring could have a role within low-risk scenarios, such as NTG monitoring. In relation to acceptability, the main facilitators included anticipation that home monitoring may increase healthcare capacity, tackle issues regarding physical access to clinic and the belief that inadequate alternatives may currently be present. The main barriers predicted by participants included a lack of clinician trust in equipment, fear of patient harm and physical patient ability issues. Our findings suggest several challenges need to be overcome to achieve clinician buy-in and to successfully integrate glaucoma home monitoring into care.

### Strengths and limitations of study

The survey had a good estimated response rate (68%) and the inclusion of free-text responses allowed collection of richer data. However, a limitation of this study was the evidence of survey fatigue. The initial demographics and scenario 1 response rate was at 100% (n=49), which then dropped to 88% (n=43) by scenario 3. This suggests that the scenarios may have been tiresome and that the results collated may not be fully representative of all views (due to a progressive lack of engagement).

Another limitation includes that the survey design did not have a question to differentiate whether participants were responding from UKEGS or from other sources (such as through our clinical PIs networks), limiting our ability to determine the source of respondents. However, the survey did include initial screening questions to ensure that only specialist respondents directly involved in glaucoma care could proceed forward.

### Strengths and limitations of study in relation to other studies

No consensus between specialists regarding the ideal patient for home monitoring was identified. Some participants anticipated that low-risk patients would be suitable due to stable disease and consequent low risk of missing progression. However, others felt that using home monitoring of low-risk patients would not be resourceful. Across scenarios, there were many concerns about anticipated patient-related issues inhibiting the patient’s ability to use home-monitoring technologies. However, previous findings suggest that patients, in the typical demographic impacted by glaucoma, have had high compliance rates with digital health technologies.^5 22 23^ This may reflect the self-selection of patients previously involved in these studies as they have demonstrated motivation and enthusiasm for monitoring technologies, which may not be representative of typical patients, despite some studies indicating similar median participant ages to the average glaucoma patient.^5^ However, this could equally suggest clinicians underestimate the ability and acceptance patients have for technologies. Greater understanding technology application and determining ideal patient selection is essential for the future development.

Participants anticipated that integrating home monitoring into the current system could act as an adjunct, increasing hospital capacity for patients who require face-to-face assessment. This potential benefit may be significant as services are currently struggling to meet the demand for care.^24^ The rationale behind home monitoring could allow low-risk patients to be efficiently assessed remotely, therefore, reducing clinic footfall and increasing hospital capacity for high-risk patients. Therefore, integration of home monitoring could support timely patient care, promoting patient safety and overall encourage greater rates of assessment. This may additionally optimise human and financial resources to maximise service capacity.

The subtheme of ‘Clinician Trust in Equipment’ was highlighted as area of concern for participants, and at least partially attributable to minimal participant exposure to home-monitoring devices. Many participants expressed that their judgement regarding home monitoring may change if they could be reassured through evidence. While some evidence suggests that iCare tends to overestimate/underestimate pressure measurements within 0–5 mm Hg of the actual pressure,^16^ many others remain confident that the results are comparable to hospital gold standards and identified variations clinically acceptable.^13^,^18^ Measurement variation may be attributable to factors such as circadian rhythm, causing IOP changes throughout the day.^13^ Our findings suggest low confidence in device reliability resulting in clinicians being hesitant to adopt these technologies at present. Further evaluation using a comparative study between hospital and iCare tonometers within the same time frame to assess pressure discrepancy, and effective communication of these findings with clinicians, will be key to overcoming this challenge.

### Future research

This study has demonstrated a requirement for further research to determine the ideal use of this technology:

1. Defining precisely the target patient population. A clear purpose for the technologies needs to be agreed on prior to defining what patient parameters could be used to include/exclude for home monitoring.
2. Quantifying the proportion of patients and types of patients who would be able to use this technology.
3. Identifying what educational needs and training may be required to support patients using the technology.
4. Determining optimum frequency and duration of use of these technologies in routine clinical practice.

Future research exploring how to overcome the concerns perceived by clinicians could be conducted through qualitative methods to allow detailed exploration of views and allow opportunity to clarify any vague responses, ideally generating a consensus on when technologies would be useful. Future studies should also involve specialists working in community settings, where glaucoma monitoring also frequently occurs, to further understand implementation issues.

## Conclusion

Overall, utilisation of hypothetical patient scenarios within this study has identified that there is limited agreement among clinicians regarding which glaucoma patients are most suitable for home monitoring using digital technologies to measure IOP and VFs. Of all scenarios, the highest degree of alignment (>60%) was demonstrated for scenario 4 (a stable, low-risk patient) in support of IOP and VF home monitoring. Despite this, clinicians anticipated concerns regarding home monitoring of high-risk patients, due to the fear of missing progression or unreliable readings. However, clinicians reported that home monitoring could play a key role within low-risk scenarios such as NTG and 24-hour phasing. Overall, clinicians reported that integrating home monitoring into our current system could act as an adjunct to promote hospital capacity for high-risk patients who require face-to-face appointments and provide an adequate solution to the continuously increasing glaucoma caseload.

However, further research is required to address the barriers identified, including an evaluation of device reliability to promote clinician confidence in equipment. Clinicians expressed concerns about patient safety, decreased glaucoma progression detection and concerns about how resourceful this approach could be in comparison to current provision. Our findings identify important questions requiring further exploration, underlying the significance and value of this study.

## supplementary material

10.1136/bmjopen-2023-080873 online supplemental figure 1

10.1136/bmjopen-2023-080873 online supplemental file 1

## Data Availability

No data are available.
