# Supplementary figures and images for: Which glaucoma patients should be monitored at home and exploration of clinician perceptions on home monitoring? a survey of glaucoma specialists in the UK
Source: BMJ Open. 2024 Nov 18;14(11):e080873. doi: 10.1136/bmjopen-2023-080873 (PMC11574396; doi:10.1136/bmjopen-2023-080873)

**Supplementary Figure 1: Summary Graph of Participant Responses (n=49)**

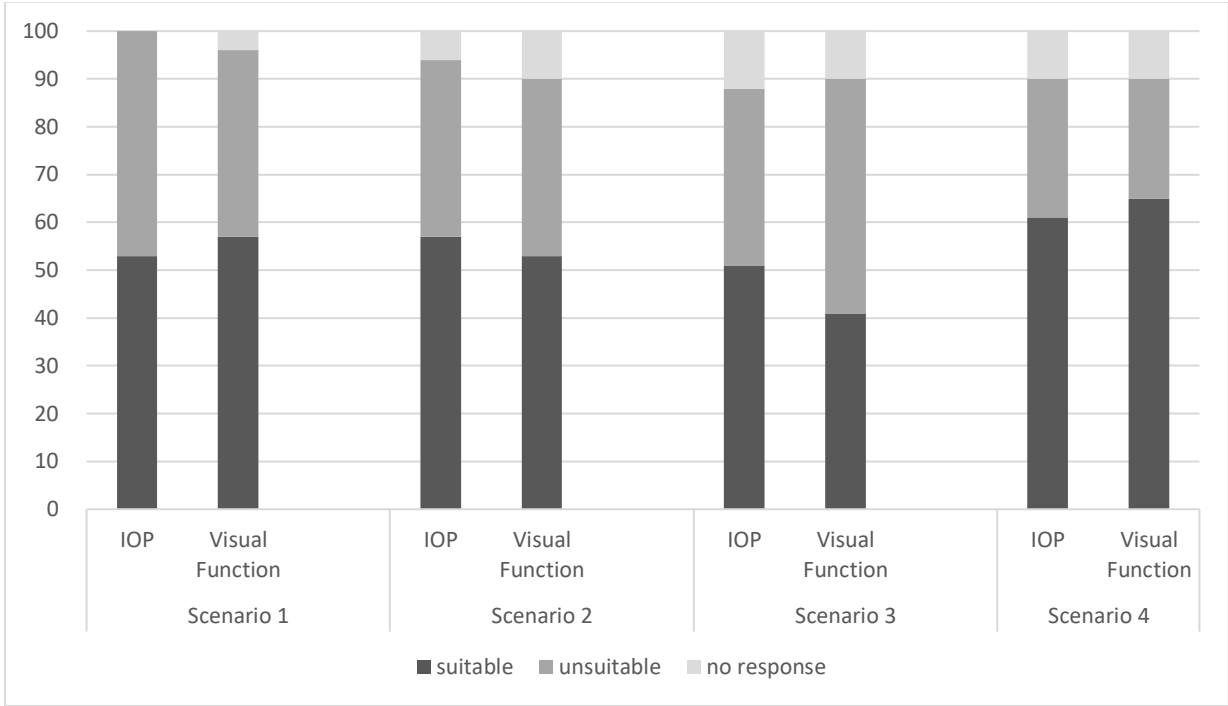

Supplement: online supplemental figure 1 [file bmjopen-14-11-s001.pdf]
