# Supplementary material for: Which glaucoma patients should be monitored at home and exploration of clinician perceptions on home monitoring? a survey of glaucoma specialists in the UK
Source: BMJ Open. 2024 Nov 18;14(11):e080873. doi: 10.1136/bmjopen-2023-080873 (PMC11574396; doi:10.1136/bmjopen-2023-080873)
Supplement: online supplemental file 1 [file bmjopen-14-11-s002.pdf]

Alagappan et.al. (2023): Which Glaucoma Patients Should Be Monitored at Home: A Survey of Glaucoma Specialists in the UK. - **Supplementary Information**

Table of Contents

1. Study Protocol 2

2. Original Survey 12

3. Supplementary Table 1: Coding Dictionary 29

4. Supplementary Table 2: Suitability Summary 38

5. Supplementary Table 3: Frequency and Duration Summary 39

1. Study Protocol

Full Title:	In-home Tracking of glaucoma Work Package 1: which patients are most appropriate for self-monitoring?
Study Acronym:	**I-TRAC WP1**
Sponsor:	University of Aberdeen
Sponsor Reference Number:	Not applicable
Funder:	National Institute for Health Research, Health Technology Assessment Programme
Chief Investigator:	Dr Katie Gillies
REC Reference Number:	Not applicable
R&D Reference Number:	Not applicable
ISRCTN / Clinicaltrials.gov No:	Not applicable
Version Number and Date:	Version 2

Protocol Approval

Home monitoring for glaucoma: which patients are most appropriate for self-monitoring?

Signatures
By signing this document I am confirming that I have read, understood and approve the protocol for the above study.

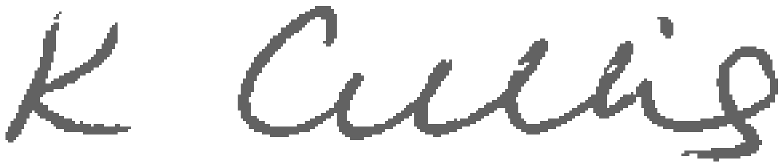

Dr Katie Gillies		11th August 2020
Chief Investigator	Signature	Date

Version	Date	Change
1	180820	Approval of V1
2	210920	Change to also include the UK & Eire Glaucoma Society (UKEGS) in survey.

List of Abbreviations

Compile a list of abbreviations as appropriate.

CI	Chief Investigator

CNORIS	Clinical Negligence and Other Risks Scheme
CRF	Case Report Form
DMC	Data Monitoring Committee
GCP	Good Clinical Practice
ISF	Investigator Site File
PI	Principal Investigator
PMG	Project Management Group
R&D	Research and Development
REC	Research Ethics Committee
SOP	Standard Operating Procedure
TMF/SMF	Trial/Study Master File
TSC	Trial/Study Steering Committee
Other	

Summary

Glaucoma is a common chronic eye condition and the second commonest cause of blindness in the UK. It is typically influenced by the pressure in the eye (intraocular pressure) being too high, for a particular person. Glaucoma impairs mainly the peripheral vision (visual field). Treatments reduce eye pressure to delay or stop glaucoma getting worse. However, in some glaucoma may still progress, so patients need regular monitoring at hospital eye services where they have their eye pressure and the visual field measured. This allows doctors to assess effectiveness of current treatment and detect glaucoma progression. Patients need these check-ups for the rest of their lives.

Hospital eye services are very busy, accounting for 10% of all NHS outpatient visits. Glaucoma patients represent a significant part of this workload, in England alone over 1 million visits per year are for glaucoma patients. Providing regular surveillance and treatment is already a major challenge for the NHS. The prevalence of glaucoma increases with age. Demand for glaucoma care is increasing (and will continue to do so) due to our aging population.

Recent advances in technology mean it is now possible for glaucoma patients to monitor eye pressure and visual fields in their own home. Their information could be transferred to the hospital for interpretation by a health care professional, or they could request hospital appointment if the home tests show their glaucoma has worsened or eye pressure has increased. Home monitoring could mean patients requiring fewer hospital check-ups, whilst increasing convenience and potentially reducing costs and increase capacity for the NHS.

Currently though, we do not know if home monitoring is acceptable to people with glaucoma, or if home monitoring in the general glaucoma population is feasible. The main aim of our study is to assess acceptability and feasibility of home monitoring, and to make recommendations about future research to test how the NHS could use home monitoring.

The project detailed in this protocol outlines the first work package (WP1) of a multiphase project. Work package 1 aims to identify which patients would be most appropriate for home monitoring. Frequency of monitoring for glaucoma patients is determined by severity of disease and rate of progression. At the moment it is uncertain which population would benefit most from home monitoring, e.g. those with stable and well controlled disease or those with severe glaucoma at high

risk of progression. This first WP will then inform the subsequent phases of the project, which will involve NHS patients and is being reviewed by an NHS REC.

This research fits one of the top five research recommendations by the James Lind Alliance, i.e., "What can be done to improve early diagnosis of sight-threatening glaucoma"? We have included a patient as independent member of the Study Steering Committee who will be actively involved in the conduct and governance of the research. We will also involve the International Glaucoma Society in an advisory role. Results of the study will be shared with those who participated and with relevant stakeholders in Hospital Eye Services.

1.	**Introduction**
1.	**Background**
	Resource constraints resulting in delays in patients' access to glaucoma services have resulted in vision loss due to glaucoma [1]. Glaucoma services are overwhelmed and struggling to accommodate current demands [8]. Reducing the need for hospital based services will improve the ability to see those most at risk of vision loss, which could alleviate both demand on the service and improve patient outcomes. Digital technologies that provide opportunities for home monitoring of glaucoma progression have potential to contribute to solve these challenges and, potentially, improve outcomes. However, understanding which patients could benefit most, the acceptability of the technologies, and the implications for the service need to be resolved before a definitive evaluative study can be conducted. The feasibility study outlined in this application will address these uncertainties.<br><br>There are recent advances for home monitoring of chronic diseases such as type 1 diabetes (e.g., real-time continuous glucose monitoring, where glucose levels can be accessed electronically by physicians) and high blood pressure (ambulatory blood pressure monitoring, ABPM).<br><br>Glaucoma is the second leading cause of blindness in the UK and it is potentially preventable. Glaucoma is an age-related chronic and progressive eye condition that requires regular monitoring at hospital eye services (HES). When diagnosis of glaucoma is confirmed, treatment with anti-glaucoma therapy is started. Treatment is escalated when there is a diagnosis of progression of disease, typically with visual field testing, or when the intraocular pressure (IOP) is above the individualised target level. When patients receive additional treatment (e.g., additional eye drops) patients are reassessed at each subsequent visit to determine disease stability and IOP control, to decide whether further treatment escalation is necessary.<br><br>Hospital eye services (HES) account for 10% of the NHS outpatient activity, and about ¼ of all outpatient visits to HES are due to glaucoma. Thus monitoring of patients with glaucoma generates a considerable burden for the NHS and for patients. Over £500m is currently spent on glaucoma care in the NHS [9]. This is likely to increase as the population ages and more people develop glaucoma during their lifetime and require longer periods of monitoring as they live longer [8]. Already there is evidence that burden of glaucoma follow-up on the NHS is exceeding resources to undertake it

	and there is UK-wide data that lack of timely monitoring has resulted in some glaucoma patients losing vision and even progressing to blindness [1]. Evaluations of digital technologies (such as apps) for home monitoring to reduce demand on the service whilst simultaneously improving patient outcomes through earlier detection of disease progression are an urgent priority.
2.	**Rationale for Study**
	The need for this research is multipronged and addresses calls from national funders, the Department of Health, and the James Lind Alliance. The work described in our proposal will generate evidence on the feasibility of home monitoring for glaucoma and whether the use of digital technologies in this context have the potential to improve efficiencies for the NHS and self-management for patients.<br><br>Digital technologies are now available for regular monitoring of glaucoma by patients at home. Specifically, applications for self-monitoring of visual function (visual field test) and the Icare HOME technology, which has been developed to measure IOP at home. These technologies are safe, FDA approved and CE marked, and allow data to be acquired at home and potentially transmittable to a hospital without the need for patients to interpret tests results, making home monitoring of glaucoma practicable.<br><br>In a new model of care implementing digital technologies in this setting, glaucoma patients would be monitored using the home monitoring tests rather than attending HES. If the tests confirmed that glaucoma is under control further HES visits would not be needed. If the home monitoring tests indicated a deterioration, the patient or the clinician would arrange an appointment and/or a prescription for additional treatment would be issued. Under this new model, the focus of NHS hospital glaucoma clinics would then shift to providing appointments to people with progressing or uncontrolled disease, rather than regular monitoring of patients with good disease control. This shift would allow amplifications in staff productivity by releasing time previously committed to regular monitoring appointments. However, before the benefits of digital technologies for glaucoma home monitoring are realised the feasibility of their use in practice and the potential benefits for patients and the health care service needs to be assessed.<br><br>The project detailed in this protocol outlines the first work package (WP1) of a multiphase project. Work package 1 aims to identify which patients would be most appropriate for home monitoring. Frequency of monitoring for glaucoma patients is determined by severity of disease and rate of progression. At the moment it is uncertain which population would benefit most from home monitoring, e.g. those with stable and well controlled disease or those with severe glaucoma at high risk of progression. In order to identify which patents may be most appropriate for home monitoring we will use vignettes describing different clinical scenarios covering patients with low and high risk of disease progression. Clinical vignettes are simple, efficient tools used to measure variation in clinicians' beliefs, attitudes and behaviours in relation to diagnosis and management of

	patients with similar conditions . This first WP will then inform the subsequent phases of the project, which will involve NHS patients and is being reviewed by an NHS REC.
2.	**Study Objectives**
1.	**Objectives**
1.	**Primary Objective**
	Identify which glaucoma patients are most appropriate for home monitoring (e.g. all patients, or those with stable disease, or those with severe glaucoma?);
2.	**Secondary Objectives**
	Not applicable
2.	**Outcomes**
1.	**Primary Outcome**
	Clinical parameters of glaucoma patients eligible for home monitoring
2.	**Secondary Outcomes**
	Not applicable for research question and study design.
3.	**Study Design**
1.	**Study Description**
	An online survey to identify glaucoma patients most suitable for home monitoring will be hosted and disseminated through the Survey Monkey platform. Consultants Ophthalmologists who are members of the Royal College or the UK & Eire Glaucoma Society (UKEGS) will be sent an invitation to participate in the study with a weblink to the survey through the Royal College Ophthalmologists (RCOphth) and the UKEGS distribution lists.<br><br>The survey will ask clinicians to consider a variety of scenarios or vignettes. In this case the vignettes will take the form of brief narratives containing key items of information about glaucoma severity (mild, moderate, severe), current treatment, disease control (apparently well controlled, uncertain) and management options that are available to fictional patients. These vignettes will be developed by the Research Fellow through discussion with the three clinical leads for each of the recruiting sites in subsequent phases of the project.<br><br>The order of the presentation of the vignettes will be randomised and presented. For each clinical vignette, Consultant Ophthalmologists will be asked to consider whether these patients would be appropriate for home monitoring using the digital technologies being assessed in this application. The data from the clinical vignette will be presented and the consultants will be asked to score a patient as either 'Appropriate'/'Not appropriate'/'Unclear' for home monitoring. If 'unclear' is selected further information will be requested (through open text comments boxes) for justification of this response.
2.	**Study Flowchart**

	Not applicable
3.	**Study Matrix**
	Not applicable
4.	**Study Population**
1.	**Number of Participants**
	The survey will be hosted and disseminated through the online Survey Monkey platform . Consultants Ophthalmologists who are members of the Royal College or the UKEGS will be sent an invitation to participate in the study with a weblink to the survey through the Royal College Ophthalmologists (RCOphth) and UKEGS distribution lists. The RCOphth and UKEGS distribution lists have previously been utilised for disseminating surveys for research purposes and the RCOphth have agreed to disseminate our survey. The RCOphth list can facilitate dissemination to approximately 100 clinical lead Consultant Ophthalmologists with a predicted response rate based on other RCOphth administered surveys of 45%. The UKEGS will enrich the sample for Consultant Ophthalmologists who are currently specialising in treating patients with glaucoma. We aim to recruit a suitable number of Consultant Ophthalmologists who are representative of those working in the UK and making decisions about the clinical care of patients with glaucoma.
2.	**Inclusion Criteria**
	Consultant Ophthalmologists who currently treat patients with glaucoma and are members of and included on the distribution list for the Royal College of Ophthalmologists.
3.	**Exclusion Criteria**
	There are no specific exclusion criteria
5.	**Participant Selection and Enrolment**
1.	**Identifying Participants**
	Consultants Ophthalmologists who are members of the Royal College or UKEGS will be sent an invitation to participate in the study with a weblink to the survey through the Royal College Ophthalmologists (RCOphth) and UKEGS distribution lists. The RCOphth and UKEGS distribution list has previously been utilised for disseminating surveys for research purposes
2.	**Consenting Participants**
	As per the Heath Research Authority guidance on seeking consent for online surveys, consent is implicit by completion and return of the questionnaire. Therefore consent is contingent on completion and submission of the online questionnaire but explicit written consent is not required.
3.	**Screening for Eligibility**
	Not applicable
4.	**Ineligible and Non-Recruited Participants**
	Not applicable
6.	**Randomisation and Blinding**
1.	**Randomisation Details**

	Not applicable
2.	**Blinding**
	Not applicable
3.	**Withdrawal Procedures**
	Whilst it is unlikely that participants will withdraw from this one-off online survey, in the event that they wish to do so they will be made aware that if they withdraw consent the data collected to date will still be used (anonymously) in the analysis. The only withdrawal criteria for the study would be that a participant does not regularly treat patients with glaucoma.<br>If a participant is required to be withdrawn from the study (either through study exclusion or withdrawal of consent), aggregate level data on reason for exclusion will be collected, no direct efforts to replace individuals will be made, and data will be retained with the appropriate permissions as detailed in the PIL.
7.	**Study and Safety Assessments**
	This phase of the research is a staff only survey about identification of appropriate glaucoma patients who would be eligible for home monitoring. It does not raise any substantial safety issues.
8.	**Data Collection and Management**
1.	**Data Collection**
	Data will be collected through the online Survey monkey platform. Data downloads from the platform will be stored securely on a password protected shared drive on a University of Aberdeen server.
2.	**Data Management System**
	Participants will be assigned a unique identifier on their questionnaire. All electronic resources will be stored on the University of Aberdeen server, with access restricted to the study team.
9.	**Labs and Samples Analysis**
1.	Not applicable
10.	**Statistics and Data Analysis**
1.	**Sample Size Calculation**<br>No formal sample size has been calculated as the purpose of this survey is expert opinion. The sample will be a convenience sample generated from the membership of the Royal College of Ophthalmologists.
2.	**Proposed Analysis**
	Data will be analysed using descriptive statistics and reported using frequencies to identify which cases there is most agreement as being appropriate for home monitoring. Vignettes for which there is more than 50% of respondents listing as 'Unclear', analysis of the free text will be conducted using a content analysis approach to determine whether and how the patients presented in these vignettes could be suitable for home monitoring.
3.	**Missing Data**
	Not applicable

4.	**Transfer of Data**
	Not applicable
11.	**Trial/Study Management and Oversight Arrangements**
1.	**Trial/Study Management Group**
	The overall multi-stage study will be co-ordinated by a Study Management Group, consisting of the grant holder (CI), coapplicants, external PIs, PPI partner, and Research Fellow.
2.	**Trial/Study Management**
	A Research Fellow will oversee the study and will be accountable to the CI. The Research Fellow will be responsible for checking the completeness, plausibility and consistency of the data. However, this remains the overall responsibility of the CI. Any queries will be resolved by the CI or delegated member of the study team.
3.	**Trial/Study Steering Committee**
	An independent Study Steering Committee (SSC) will be established to oversee the conduct and progress of the entire study as per the recommendations from the funder.
4.	**Data Monitoring Committee**
	An independent Data Monitoring Committee (DMC) is not required for this study as confirmed by the funder.
12.	**Inspection of Records**
**12.1**	The CI, PIs and all institutions involved in the study shall permit study related monitoring, audits, and REC review. The CI agrees to allow the Sponsor or, representatives of the Sponsor, direct access to all study records and source documentation.
13.	**Good Research Practice**
1.	**Ethical Conduct of the Study**
	The study will be conducted in accordance with the principles of good clinical practice (GCP).<br>In addition to Sponsorship approval, a favorable ethical opinion will be obtained from the appropriate REC prior to commencement of the study.
1.	**Confidentiality**
	All records will be identified in a manner designed to maintain participant confidentiality. All records will be kept in a secure storage area with limited access to study staff only.<br><br>The CI and study staff involved with this study will not disclose or use for any purpose other than performance of the study, any data, record, or other unpublished, confidential information disclosed to those individuals for the purpose of the study. Prior written agreement from the Sponsor or its designee will be obtained for the disclosure of any said confidential information to other parties.

2.	**Data Protection**
	The study team involved with this project will comply with the requirements of the General Data Protection Regulations (GDPR) and the Data Protection Act 2018. The HRA recommended wording to fulfil transparency requirements under the GDPR for health and care research will been included in the survey.<br>The CI and study staff will also adhere, if appropriate, to the current version of the NHS Scotland Code of Practice on Protecting Patient Confidentiality. Access to collated participant data will be restricted to the CI and appropriate study staff.<br>Computers used to collate the data will have limited access measures via user names and passwords.<br>Published results will not contain any personal data that could allow identification of individual participants.
3.	**Insurance and Indemnity**
	The University of Aberdeen is sponsoring the study.<br><br>**Insurance**<br><br>• The University of Aberdeen will obtain and hold a policy of Public Liability Insurance for legal liabilities arising from the study.<br><br>**Indemnity:** The Sponsor does not provide study participants with indemnity in relation to participation in the Study but has insurance for legal liability as described above.
14.	**Study Conduct Responsibilities**
1.	**Protocol Amendments, Deviations and Breaches**
	The CI will seek approval for any amendments to the Protocol or other study documents from the Sponsor (in the first instance), REC and NHS R&D Office(s). Amendments to the protocol or other study documents will not be implemented without these approvals.<br>In the event that a CI needs to deviate from the protocol, the nature of and reasons for the deviation will be recorded in the CRF, documented and submitted to the Sponsor. If this necessitates a subsequent protocol amendment, this will be submitted to the Sponsor for approval and then to the appropriate REC and lead NHS R&D Office for review and approval.<br>In the event that a serious breach of GCP is suspected, this will be reported to the Sponsor immediately using the form “Breach Report Form”.
2.	**Study Record Retention**
	Study documents will be retained for 5 years after study end date.
3.	**End of Study**
	The end of study is defined as completion of data analysis and study reporting. The Sponsor, CI and/or the TSC have the right at any time to terminate the study for clinical or administrative reasons.

	The end of the study will be reported to the Sponsor and REC within 90 days, or 15 days if the study is terminated prematurely. The CI will ensure that any appropriate follow up is arranged for all participants.<br>A summary report of the study will be provided to the Sponsor and REC within 1 year of the end of the study.
15.	**Reporting, Publication and Notification of Results**
1.	**Authorship Policy**
	Ownership of the data arising from this study resides with the study team and their respective employers. On completion of the study, the study data will be analyzed and tabulated, and a clinical study report will be prepared.
2.	**Publication**
	The study report will be used for publication and presentation at scientific meetings. Investigators have the right to publish orally or in writing the results of the study.<br>Summaries of results will also be made available to Investigators for dissemination within their clinical areas (where appropriate and according to their discretion).
3.	**Peer Review**
	We will not seek an additional internal review prior to submitting for CERB approval. This work package has already been externally peer reviewed within an application for the bigger project as part of the National Institute for Health Research funding process and this documentation will be submitted as part of the CERB application.

2. Original Survey

Participant information leaflet

What is the purpose of this study?

Recent advances in technology mean it is now possible for glaucoma patients to monitor eye pressure and visual function in their own home. This online questionnaire, which has been informed by current evidence and guidelines for glaucoma care, aims to identify which glaucoma patients would be most appropriate for home monitoring using this technology. At the moment it is uncertain which population would benefit most from home monitoring, e.g. those with stable and well controlled disease or those with severe glaucoma at high risk of progression. Hence, survey will provide information about which population would be most appropriate to monitor with this technology which will directly feed into the next stage of the project – inviting patients to use the technology to monitor their glaucoma at home and assessing the feasibility and acceptability of doing so.

Do I have to take part?

It is your decision about whether or not you wish to take part. If you do agree to take part and then change your mind, you can withdraw at any time without giving a reason however, the data you have provided to that point would still be included in any analysis.

What happens next?

If you would like to take part, please click the 'next' tab at the bottom of this page which will take you directly to the questionnaire. The questionnaire will take no longer than 30 minutes to complete. All information which is collected about you during the course of this study will be kept strictly confidential. If you agree to participate in this study, you may be asked to participate in other projects linked to this research.

There will be no extra benefit to you if you do take part in the study but by doing so you will be helping with this research. We do not anticipate there to be any risks associated with participating in this research.

What will happen to the results of the study?

We will use the results of this study to help make decisions about future research in this area, specifically which patients with glaucoma we should invite into the next stage of the feasibility project. The researchers may also report the findings in a scientific journal and at a scientific research meeting. The information that we report would be completely anonymous and would not identify you in any way.

What ethical and data permissions are in place?

This study has been reviewed and received favourable opinion by the University of Aberdeen School of Medicine, Medical Sciences and Nutrition's College Ethics Review Board . All electronic data collected for the purpose of the research study will be confidentially and securely stored on computer servers maintained by the University of Aberdeen. The study team or other individuals from the University of Aberdeen may look at data collected for the study, to check that the study is being carried out correctly and to check the accuracy of the research study. The University of Aberdeen is the controller for this study and is responsible for looking after your information, using it properly and complying with your rights. You can find more about this at www.abdn.ac.uk/privacy or by contacting us at the address below.

Whom do I contact if I have a concern or a complaint?

If you have a concern about any aspect of this study, you should ask to speak to the researcher (Dr Carrie Stewart) or Chief Investigator (Dr Katie Gillies) who will do their best to answer your questions [carrie.stewart@abdn.ac.uk; k.gillies@abdn.ac.uk]. If you remain unhappy and wish to complain formally,

you can do this by contacting the Research Governance Team by emailing researchgovernance@abdn.ac.uk or by calling 01224 551123.

Do you provide care for patients with glaucoma as part of your regular clinical activity?
Yes No

<If No, survey ends on disqualification page>

Section 1 Questions about you and your practice

1. Please select which job title best describes your current employed position:

Consultant ☐ Associate Specialist☐ Other ☐

2. Please select which option best reflects how long you have been treating patients with glaucoma:

<5 years☐ 5-10 years ☐ >10 years ☐

3. Please select which option reflects your age:

<40☐ 40-49☐ 50-59☐ 60+☐

4. Please select which option reflects your gender identity?

Male☐	Female☐	Non-binary☐	Gender-fluid☐	Agender☐
Prefer not to say☐	Prefer to self-describe: ☐	Please describe:		

5. Please select which option best reflects your ethnicity:

**White**	**Mixed/ Multiple Ethnic Groups**	**Asian/ Asian British**	**Black/ African/ Caribbean/ Black British**	**Other Ethnic Group**
English ☐	White and Black Caribbean ☐	Indian ☐	African ☐	Arab ☐
Welsh ☐	White and Black African ☐	Pakistani ☐	Caribbean☐	Any other ethnic group ☐
Scottish☐	White and Asian ☐	Bangladeshi☐	Any other Black/ African/ Caribbean background☐	
Northern Irish☐	Any other mixed/ multiple ethnic background ☐	Chinese ☐		

British☐

Irish☐

Romani or Irish traveller☐

Any other white background☐

Any other Asian background☐

6. Are you currently using a device to measure patients' IOP / VF at home? If yes, which device(s)

Yes ☐ No ☐

If Yes, please tell us the name of the devices that you are using:

7. If using a device for measuring IOP, can you provide an example(s) of for whom/in what circumstances?

8. If not using a device, can you suggest when using one might be useful?

9. Are you currently using a device to measure patients' VF at home? If yes, which device(s)

Yes ☐ No ☐

If Yes, please tell us the name of the devices that you are using:

10. If using a device for VF testing at home, can you provide an example(s) of for whom/in what circumstances?

11. If not using a device, can you suggest when using one might be useful?

Section 2 Evidence-based monitoring of glaucoma:
We would like to summarise below the key NICE recommendations for monitoring of people with OHT and glaucoma that may be useful when you review the clinical vignettes in section 3.

National Institute for Health and Care Excellence (NICE). Glaucoma: diagnosis and management NICE Guideline 81. Oct 2017. Available: https://www.nice.org.uk/guidance/ng81/evidence/full-guideline-pdf4660991389 [Accessed 1st March 2021]. Management and monitoring of OHT and POAG according to NICE 2017

1. Management and monitoring of OHT according to NICE:

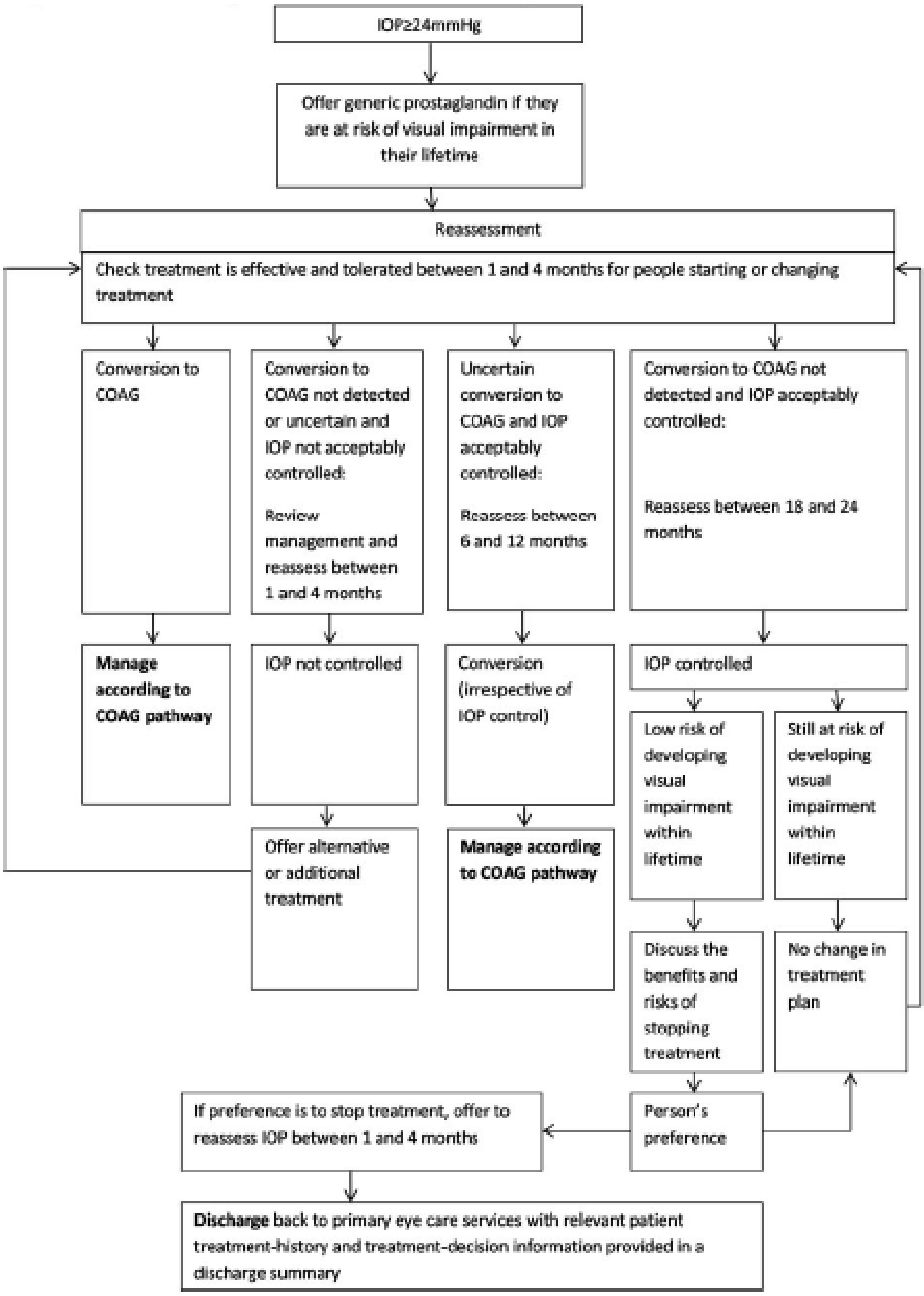

2. Monitoring of POAG according to NICE

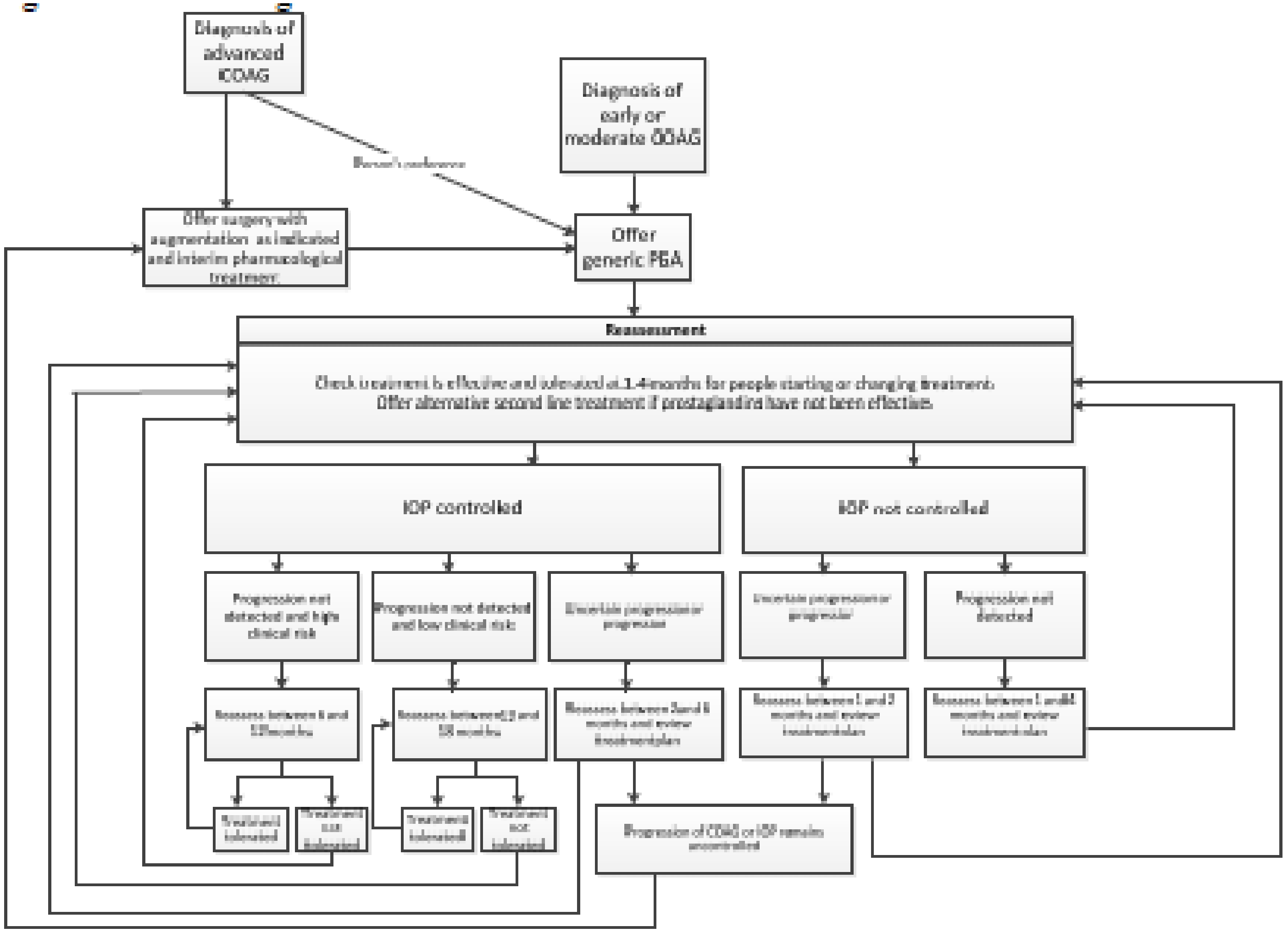

We are exploring the use of two technologies for monitoring glaucoma at home. These technologies are:

(1) iCare HOME tonometry

[Example image of black male using iCare tonometer device]

Icare HOME (Figure above) is a handheld rebound tonometer that has an automatic-side recognition and positioning assistant for the correct alignment of the tonometer. During measurement, Icare Home is placed at 4 to 8 mm from the cornea where the inbuilt software acquires 6 readings. The highest and lowest IOP reading are eliminated, leaving four readings which are be averaged and displayed on the device. If reliable readings are not obtained, an error bar is displayed and patients will need to repeat the measurements. The Icare HOME stores the final IOP with other information such as date, time, laterality of the eye, and measurement quality into its built-in memory module. Icare HOME can be linked to the patient's smartphone and data can be uploaded and retrieved from the cloud allowing remote access to the data. Home tonometry can be used by the patient or by another person.

Published literature on iCare HOME has reported moderate agreement between Icare HOME and GAT in glaucoma patients. It seems most patients are able to measure their own IOP at home after given appropriate training and instruction.

(2) A tablet-based app for measuring Visual Function

There are several portable applications now available to measure different aspects of visual function including visual field, visual acuity, and contrast sensitivity. These include EyeCatcher, MRF and OKKO Health.

Results of these tests can be downloaded to a cloud-based file that can be accessed by the clinician.

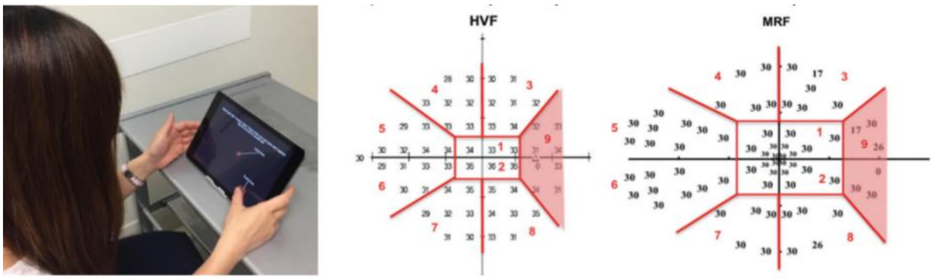

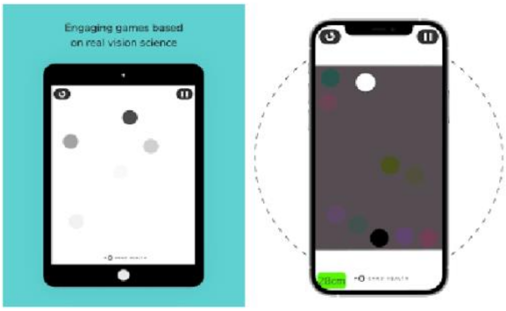

Top Figure: Left: patient using the MRF on a tablet, stimuli are shown in various locations on the screen and the patient taps when they are seen. Right: Stimuli comparison between HVF and MRF

Bottom figure: Example screens of visual function tests using OKKO Visual Health AppResults of MRF are downloaded to a cloud-based file that can be accessed by the clinician.

Section 3 Clinical Vignettes
We would now like you to read each of the five clinical scenario's provided and consider what you would do in each. Please assume that the technologies will be available for all at no cost to the patient.

1. **The first patient** is a 63 year old White male (Mr Smith) with severe glaucoma in both eyes (MD -15 dB RE and -19 dB LE), first diagnosed 2 years ago. Presenting IOP was 25 / 26 mmHg and CCT 534 /543. IOP is now 18-19 mmHg on two eye drops, and since diagnosis he has had 3 visual field tests and five clinic visits. There is no evidence of progression in VF and OCT. He seems reluctant to consider surgery.

1.1 Would you consider using the iCare HOME tonometry test?	Yes☐	No ☐

**If yes:** How frequently would you ask Mr Smith to take measurements? Would you ask him to measure IOP at different times?	
**If yes:** For what duration would you ask Mr Smith to use this test before reassessing in the hospital?	
**If no:** Why would you not consider Mr Smith for iCare HOME tonometry test?	

1.2 Would you consider it useful to monitor the visual function at home using a visual function application?	Yes☐	No ☐

**If yes** how frequently would you ask Mr Smith to do visual function testing at home?	

**If yes** for what duration would you ask Mr Smith to use this test before reassessing in the hospital?	
**If no:** Please explain why you would not find it useful to monitor the visual function at home for Mr. Smith?	
Would your opinion change if Mr Smith had the same clinical parameters but was 83 years old?	

2. **The second patient** is a 70 year old White female (Ms Adams) with OHT in both eyes (normal VFs, normal RNFL OCT, large optic discs but no signs of glaucoma) diagnosed last year. Presenting IOP was 28 mmHg BE, and CCT 550/545. After treatment with latanoprost her IOP is 23 mmHg.

2.1 Would you consider using the iCare HOME tonometry test?	Yes☐	No ☐

**If yes:** How frequently would you ask Ms Adams to take measurements? Would you ask her to measure IOP at different times?	
**If yes:** For what duration would you ask Ms Adams to use this test before reassessing in the hospital?	

**If no:** Why would you not consider using the iCare HOME tonometry test for Ms. Adams?	

2.2 Would you consider it useful to monitor visual function at home with a visual function application? Yes☐ No ☐

If yes how frequently would you ask Ms Adams to do visual function testing at home?	
If yes for what duration would you ask Ms Adams to use this test before reassessing in the hospital?	
If no: Why would you not consider it useful to monitor visual function at homeusing a visual function application?	
What if Mrs Adams had a well controlled IOP with latanoprost, and IOP was the mid-teens, and had been stable for 6 years?	

3. **The third patient** is a 78 year-old Asian male patient (Mr Patel) with pseudoexfoliation and moderate damage in the right eye (MD -11 dB) and early glaucoma in his left eye (-4 dB LE), CCT 540/550. IOP is not well controlled (around 20 mmHg) after laser treatment and on maximum medical therapy. He was diagnosed 3 years ago. There is no evidence of OCT or visual field progression but the visual field tests are not very reliable and he finds them very tiring. No serious health problems other than he looks a bit frail.

3.1 Would you consider useful the iCare HOME tonometry test? Yes☐ No ☐

**If yes** how frequently would you ask Mr Patel to take measurements?	
**If yes** for what duration would you ask Mr Patel to use this test before reassessing in the hospital?	
**If no:** Please explain why you would not consider it useful to use the iCare HOME tonometry test for Mr. Patel?	

3.2 Would you consider it useful to monitor visual function at home using a visual function application?	Yes☐	No ☐

**If yes** how frequently would you ask Mr Patel to do visual function testing at home?	
**If yes** for what duration would you ask Mr Patel to use this test before reassessing in the hospital?	
**If no:** Please explain why you would not consider it useful to monitor the visual function at home using a visual function application for Mr. Patel?	
What if no laser treatment had occurred? What if he was ok with the visual field tests?	

4. **The fourth patient** is a Black female, Ms McEwen 55 years old, who has mild glaucoma (NTG) in both eyes (MD -3 dB RE and -5 dB LE), CCT 538/536. Maximum untreated IOP was 19 and current IOP on latanoprost is 15 mmHg. She has been followed up for 5 years and there is no progression on VF or OCT. She is having yearly follow-up visits.

4.1 Would you consider using the iCare HOME tonometry test?	Yes☐	No ☐

**If yes** how frequently would you ask Ms McEwen to take measurements?	
**If yes** for what duration would you ask Ms McEwen to use this test before reassessing in the hospital?	
**If no:** Please explain why you would not consider using the iCare HOME tonometry test for Ms. McEwen?	

4.2 Would you consider it useful to monitor visual function at home using a visual function application?	Yes☐	No ☐

If yes how frequently would you ask Ms McEwen to do visual function testing at home?	
If yes for what duration would you ask Ms McEwen to use this test before reassessing in the hospital?	
If no: Please explain why you would not consider it useful to monitor visual function at home	

using a visual function application for Ms. McEwen?	
What if she had had mild progression on VF in the last two years?	

5. **The fifth situation** is of an ophthalmologist colleague (Dr Ahmed) who does not have sufficient capacity in the clinics, and is unable to monitor patients at the recommended intervals. He has decided to concentrate on patients whose condition has progressed in the last 2 years year or have clearly uncontrolled IOP. Patients who have not progressed in the past two years and have an apparently well controlled IOP will be monitored remotely with iCare HOME a visual function application. He will review the information of these patients regularly (every 6-12 months) in "virtual clinics".

5.1 Is this an acceptable model of care for you, as a clinician? Why?	Yes ☐	No ☐
Please explain why:		
5.2 What do you think would be the advantages of this model of care?		
5.3 What do you think would be the disadvantages of this model of care?		
5.3 Can you think of any difficulties or barriers to implementing this model of care?		
5.4 Do you think that these technologies could improve monitoring of harder to reach patients with glaucoma, such as those from ethnic minority groups?		

6.1 Would you be interested in participating in a focus group to discuss the barriers and facilitators in	Yes☐	No ☐

relation to the home
monitoring of glaucoma?

If yes, please provide your email address so that we can contact you and send you further information about this study.

Thank you for taking the time to complete this survey. The results of this survey will inform a feasibility study exploring use of the two devices (iCare Home tonometer and a visual function application) as home monitoring devices for patients with Glaucoma.

End of Questionnaire

3. Supplementary Table 1: Coding Dictionary

Themes	Sub-Theme	Code	Description	Example
Resources	Divided Consensus on Financial Costs	Cost Effective	This also includes responses that this model may improve NHS expenditure, currently or in long-term.	"I am surprised that it is affordable to use an iCare HOME for these patients, I would have thought it quite expensive."<br><br>"In the long-term, it may be cheaper too"
		Poor Value	Participants believing that this model may be more expensive than alternative options available. This is explored through device cost and upkeep, staffing review costs and NHS expenses for training.	“home tonometry for all patients seems a grandiose waste of resources"<br><br>"far more cost effective to get patients to come to a local virtual clinic site."
	Human Resources Issues	Staff to train patients	Participants believing there would be an increased burden in training patients to a point of confidence in using new equipment. May require additional team members such as district nurses to aid in training (wages etc for staff, time taken)	"Training a large number of patients in use of equipment."<br><br>“Securing the funding and staffing to train patients and to troubleshoot might be a challenge”
		Staff to review home-monitoring results	Challenges with greater data set. This requires factoring in further staff and time to review the new sets of results and following up with patients via virtual consults, demanding a greater capacity than what NHS currently can offer.<br><br>Does not include mention of increased data collection benefiting the system, only when mentioning the corresponding increased levels of staff / capacity required to review the data.	"their would be a significant burden in virtually reviewing all these patients which would need to be accounted for in the business case. "<br><br>" home monitoring would need to be well supported, to train and supervise patients, and well planned, to review data"

	Divided Consensus Regarding Capacity	Increased Hospital Capacity due to intervention	Participants mentioning that this clinical model could free up space and availability within the NHS for patients who need care as a priority. Also includes mentions of optimising resources or using them better than currently. Also includes mentions of footfall becoming reduced which can enhance apacity, resources and time between appointments. This includes responses suggesting that space can be created to prioritise riskier patient groups<br><br>Can also allow monitoring between appointments which can free up resources and capacity.<br><br>Participants mention that the use of other healthcare team members (MDT) can add to this model, such as GPs and Optometrists to gather efficient data and additional imaging to support home results. This combined can reduce strain on HES and increase capacity.	"Utilitisng the limited capacity to see stable patients virtually is helpful to generate more capacity for patients who require more attention"<br><br>"Ensure pts are seen at recommended times and if uncertain results indicate possible progression can be seen in HES promptly."
		Decreased Hospital Capacity due to intervention	There may not be any improvements in capacity but instead interference due to the home monitoring interventions. This can be explored due to the clinic capacity required to review the results and organise the proposed intervention	" home monitoring would need to be well supported, to train and supervise patients, and well planned, to review data
	Better Than the Alternative of No Monitoring		The idea that some monitoring is better than the alternative of none, leading to irreversible visual loss.	"Better to get some monitoring then just being a name on the waiting list and losing sight."<br><br>"alternative is lots of patients wait excessive time with no monitoring"

	Sub-Theme	Code	Description	Example
Patient Characteristics	Divided Consensus on Patient Compliance	Improved Compliance with intervention	This covers the views that involving the patients in their own care could empower them and increase compliance. It may also balance out the risk in patients who refuse to engage with treatment at all as some monitoring is better than none. This also covers the idea that patients require high motivation levels to comply	"I am in favour of empowering and involving patients in the management of their own care and also in best utilization of resources and this model fits with that"

			Increased patient responsibility causing increased compliance also is included here, due to increased involvement in their own care.	"May empower patient and improve adherence as they get direct feedback on the effects of treatment and status of disease"
		Decreased Compliance with intervention	This sub-theme covers concerns that compliance may be an issue with the home interventions due to a variety of factors such as patient acceptance of intervention.<br><br>Participants feel that patients may not follow testing requirements correctly / have difficulty in following care instructions due to a variety of factors, including lack of motivation<br><br>Also includes concerns regarding whether a patient will try to engage and understand intervention	"The governance of non compliancy with lack of patient involvement would be another challenge"<br><br>"Patients unable to reliably perform either test should be transferred to more formal monitoring."<br><br>"patient willingness or understanding. patients not feeling supported."<br>"Patient reliability. Patients remembering to complete tests."
	Cognitive, Physical and Mental Patient Ability to preform intervention	Physical Ability	Covers dexterity, frailty and elderly age impacting a patients physical ability to carry out testing. Also includes patients physical ability to efficiently care for the home equipment<br><br>Mention of whether the patient can preform the tasks themselves due to dexterity concerns associated with ageing (target demographic for intervention)<br><br>Worries surrounding increased patient frailty corresponding with decreased suitability for home monitoring as patients may be too frail to comply. Includes mentions of co-morbidities<br><br>Mention of age not being a deciding factor but instead what can the patient do, based on driving ability, exercise tolerance and other activities.<br><br>Patient ability to perform tests correctly and have confidence in their ability and accurately as a barrier to tackle.	patient dexterity and understanding<br><br>"Patients with reduced mobility/health issues making clinic attendance or VF testing difficult."<br><br>"The ability of patients to perform this sort of testing at home and the reliability of results - this may throw up a sugnificnat number of anaomalous results meaning that these pateints are then recalled to he hospital setting"<br><br>" Will require lot of education and get confidence of the patients."<br><br>"Patients with reduced mobility/health issues making clinic attendance or VF testing difficult."

		Cognitive Ability	This includes conditions such as dementia and learning difficulities - due to correlation between elderly and dementia, participant concerns surrounding whether the patient will result in requiring additional support with care due to memory difficulty	"Forgetting the original treatment instruction"<br><br>“Care for the device by the patients”
		Mental Ability: Increased Patient Anxiety	Participants mentioning that a continuous focus on eye condition and health could cause a negative relationship with care and heightened anxiety levels. This could be amplified by patients having a lack of knowledge regarding which changes / fluctuations are worrisome.<br><br>Home monitoring may negatively place pressure on the patient to collate accurate and reliable results which could result in visual loss if preformed incorrectly. This pressure would not be present otherwise, leading to anxiety.	"They may get very anxious about small changes in results without full understanding."<br><br>"may adversely affect his quality of life due to anxiety related to the use of these test."
		Mental Ability: Alleviates Patient Anxiety	May support patients who frequently contact healthcare services currently due to heightened healthcare related anxiety. Also coded under this category if monitoring may provide ease or comfort to patient regarding using treatment correctly.	"Where they are anxious about something and have phoned in to ask for early review."

	Sub-Theme	Code	Description	Description
Clinician Confidence in Home-monitoring	Increased Clinician Confidence in Intervention	Improved Clinician Trust	Mention of clinician confidence increased due to greater quantity of collated patient data from home interventions (compared to irregular clinic readings). This model could receive readings from various points of the day to build the greater story. This will aid in better clinical outcomes and free up further clinic time if stable. Includes mentions of being able to record data before appointments.<br><br>Does not include mention of increased data collection requiring further capacity / staff to review (this is under staffing)<br><br>Includes idea that multiple tests may improve reliability and confidence in proposed intervention (as this will minimise variability)	. "More data e.g. more IOP measurements - this might allow more refined risk prediction"<br><br>"In reality glaucoma patients may actually do better with more regular IOP and field testing as will pick up discrepancies sooner and we can't to the tests this often in the clinic."<br><br>"Multiple tests balance wide test variation"

	Decreased Clinician Confidence in Intervention	Reliability Issues	This includes mentions of evidence based practice, validating devices, sensitivity and specificity concerns, false results, efficiency worries and variability potential between patients. Remarks regarding concerns on the overall reliability of these home monitoring services causing a decrease in clinician confidence regarding the proposed product. Also covering mention that in person testing would be more accurate / reliable<br><br>This is also regarding specific reliability concerns about the visual field OKKO app, clinicians mentioning a desire for further evidence. Particular worries regarding artefacts.	"We do not have enough information about effectivity. "<br><br>"buys him some time and then causes a whole load of problems with unreliable data"<br><br>"unless the VF application was shown to be as effective as formal automated perimtery and could be correlated with VF, one would be wasting old data "
		Issues in Standardising Monitoring Conditions	Within clinic, monitoring conditions can easily be managed and controlled. However, concerns expressed about standardising home conditions coded here.<br><br>Someone external may also preform tests in place of the patient, cannot monitor.	""Also, there is a possibility of someone other than the patient performing the home tests and passing it as the patients.""
		Issues with Compatibility and Consistency with hospital eye services	Expression of concern regarding how home monitoring equipment will work with (compatibility) the clinic equipment. These readings will impact treatment outcomes so further evidence regarding the accuracy level of equipment as a suitable replacement mentioned.<br><br>In particular, concerns surrounding measurement units and comparison abilities to clinic readings.	"I-care measures in clinic are sometimes higher and sometimes lower than GAT without a readily predictable pattern." "Compromise on gold standard techniques of monitoring "<br><br>"No consistency between hospital and home care tests "
		Limitations of Home-monitoring	Participants mentioning that they believe that the model proposed within the survey will not be sufficient (compared to clinic standards) as they will require further tests to assess patients properly. This includes mentions of OCT, fundal disc imaging, slit lamp exam and optic nerve head assessment. This also includes mention that the home monitoring interventions alone will not be useful in the care of patient, as they require other additional tests.	"OCT not done which may be considered important by some for early disease"<br><br>"No optic disc or slit lamp examination If the visual field is worsening there is no way to verify what the worsening field is due to."
Perceptions of Risk	Sub-Theme	Code	Description	Example

	IT Governance issues		Worries regarding the safety and upkeep of data storage and transfer using virtual platforms. These concerns are particularly regarding confidentiality and IT system failure resulting in leaks and lack of timely clinician assessment. Concerns regarding patients having a secure way to send results.	"IT works well when it works well, but more than often there are barriers and incomplete data etc, The governance of non compliancy with lack of patient involvement would be another challenge"
	Divided Consensus on Patient Safety Profile	Decreased Clinical Safety Profile	Concerns surrounding the new model negatively impacting patient safety profile due to missed progression and loss of follow up. Deterioration / progression may be missed and fluctuation may not be tracked. Co-morbidities may also be neglected. It is also a challenge to determine whether results are a patients own.<br><br>Rapport between doctor and patient plays a key role within the healthcare process, a lack of contact due to virtual monitoring ay negatively influence patient adherence and confidence within the system. It may lead to patients feeling their care is less important than face to face appointments and result in poor compliance and feelings of lack of support.	"A significant proportion of mild to moderate glaucoma with 'apparently well controlled IOP' will develop further progression of visual fields in time and this will be a permanent damage to their visual status"<br><br>"Experience tells us that some patients will lose vision in the virtual system, despite best efforts to risk stratify and see virtually."
		Increased Clinical Safety Profile	This covers the idea that the new interventions may increase patent safety profile. Mention of access to timely care, preventing blindness with regular monitoring, tracking progression before irreversible damage is done or overall improved safety and wellbeing of patients due to device. Also mentions how risk stratification can allow prioritaisation of resources to help those at high risk whilst monitoring those at low risk<br><br>Includes any mention that they would rather see patient in person. (seen within scenarios)<br><br>A wider face to face consultation allows possibility for further symptoms to be explored, extra examinations that may not have been thought to be required for care and overall better assessment.<br><br>Also worries regarding how risk will be standardised between clinicians and therefore may be negatively impacted by this intervention (due to lack of face to face contact)	"greater number of patients getting timely monitoring"<br>"Ensure pts are seen at recommended times and if uncertain results indicate possible progression can be seen in HES promptly."<br>"Improved patient safety"<br><br>"Loss of wider examination - angle status, co-morbidity"<br><br>"A significant proportion of mild to moderate glaucoma with 'apparently well controlled IOP' will develop further progression of visual fields in time and this will be a permanent damage to their visual status"
Accessibility	Code		Description	Example

	Sub-Theme	Code	Description	Example
		Language Barriers	In certain population groups, there may be issues with understanding instructions. This includes particularly ethnic minority groups. This may result in incorrect measurement's or none at all. Therefore this model may discriminate against this group who may not have the education to seek help.	"Harder to reach patients would still have a low uptake of the technology. Education is more important. Even if given device might not use or not use correctly. Pt education empowers them to access medical help"
		Disability	Patients who are impacted by psychical disabilities may not be able to undertake tests in the intended way or require extra support that may be catered for better in person. This extra support at home, via district nurse etc, may result in additional resource expenditure.<br><br>Does not include mentions of benefits of these patients being able to benefit from home care, this is mentioned under vulnerability.	"Can patient physically undertake tests,"<br>"Also, patients with physical disabilities or learning difficulties/dementia will struggle with home monitoring themselves"<br>"Difficult positioning on VF machines (sever kyphosis, bed bound)"
		Technology and Internet Access	This model may exclude those who do not have internet access or place pressure on patients to purchase these services, whereas this pressure would not have been a concern with traditional monitoring. This also applies to device access (within this study devices are given but would this be continued if implemented into monitoring schemes). Device availability is also a factor.	"internet availability for download of test results. "
Medical Risk and Suitability	Stable, Low-Risk Disease Condition	Suitable Glaucoma Disease Stages for Intervention	OHT and NTG (low risk, stable) and screening high risk patients for *developing* glaucoma<br><br>Mention of OHT patient monitoring being the target group for this monitoring model (scenario 2 responses regarding low risk / stable disease)<br>Stable, slow progression NTG diagnosis being a suitable target group for home monitoring (scenario 4)<br><br>This covers mention of high risk of glaucoma development / Screening / suspect glaucoma within response	"Only OHT patients can be managed safely with virtual clinics"<br>"Early glaucoma/OHT cases."<br><br>"Possible diurnal variation of IOP in unstable pts, new pts with NTG."<br>"NTG monitoring"<br><br>"also useful as screening test"<br>"Early glaucoma or suspect glaucoma."
		Monitoring for data collection or post care change	Covers phasing and Diagnosis Treatment change, dose change or general care change requiring extra monitoring which can be suitably managed by home monitoring during stable disease phases	"May be particularly useful immediately after diagnosis or after change in treatment to determine rate of progression"<br><br>"As a substitute for hospital phasing in NTG suspects (specially with COVID)"

			When concern regarding fluctuation of pressure so 24 hour monitoring required, improves convenience as equipment does not have to be used all day in person (for both patient and NHS). Mention of phasing / 24 hour monitoring within the answer.<br><br>Also covers mention of patients who are suspected to fluctuate greatly between different points of the days so prevents depending on unreliable results. Particularly diurnal variation.	"Patients who may be subject to significant diurnal variation."
		Low Risk of Progression	This sub-theme is split into both participants believing that this intervention would and would not be suitable for low risk / disease status patients<br><br>Unlikely patients to progress quickly due to stable condition but still require monitoring and care. Clinicians believe this will prevent unnecessary trips whilst delivering suitable monitoring and care. Also prevents missing any low-risk progression that may previously have been missed between long durations between appointments.<br><br>Includes mentions of low risk cases being both suitable and unsuitable for clinical monitoring within the clinical scenarios used in the survey. (case 2 and 4)	"Low -medium risk patients can be monitored virtually"<br><br>"Then the majority that are stable can be left out of clinic for longer. But even patients who have been stable for years can suddenly deteriorate and if then not seeing them for one year without home tests they can return with severe damage. I think this will allow early detection of change, but allow patients to stay out of clinic safely for longer"
	Unstable, High-Risk Disease Condition	Increased Disease Progression Despite Normal IOP	The target patient group being people with normal / below target IOP measurements but continue to have disease progression (may be seen through disc changes) requiring extra monitoring measures and suggested to be helped by home monitoring within the responses.	"In established NTG were progression despite good IOP in office measures."<br><br>"Patients with progressive glaucoma - with apparently "controlled" IOP"
		High risk of complications	Demanding monitoring schedule for advanced disease / high risk / rapidly progressing patients which can be aided by home monitoring to relieve some of the burden in-between F2F appointments in order to deliver timely care and utilise resources better whilst avoiding complications	"Due to the limited capacity in hospital glaucoma clinics, we should focus our resources in higher risk patients. "<br>"<br><br>"concentrating on riskier cases without losing focus on the well-controlled ones"

			Focus will remain on all patients whilst targeting in person services for those most at need with advanced disease rather than for routine appointments. Mention of prioritisation of care.<br><br>Low risk followed up virtually so creates capacity for advanced cases in person, can prioritise care for high risk without neglecting any patients. Mention of stratification or prioritisation based on severity / progression of disease.<br><br>Includes mentions of high risk cases being both suitable and unsuitable for clinical monitoring within the clinical scenarios used in the survey.(case 1 and 3)	"If the tests are reliable and sensitive enough to pick up problems such as high IOP then allowing for virtual review would enable resources to be used for at risk populations "

	Code	Description	Example
Other	Environmental Benefit	Less transport to appointments due to increased home monitoring. Less damage to environment whilst improving patient convenience mentioned. Eco-friendly, planet supporting, reduced carbon footprint.	"good for planet"<br>"Good for the planet - low carbon footprint from not having to travel to the hospital."
	Increased Patient Convenience	Mention of increased patient convenience being a benefit due to reduced frequency of F2F appointments and easing logistic difficulties that may previously have been present with traditional monitoring.	"Patient convenience"<br>"no issues with booking associated logistics with hospital appointment"<br>"More convenient for the patient"
	Physical Restrictions Inhibiting Access to Clinic	Mention of physical access to clinic barriers being tackled including: physical travel distance to HES, domically home care benefiting from not having to attend in person, comorbidities preventing travel, care home residents unable to attend clinic, lockdowns.	"Bedbound patients in care homes"

4. Supplementary Table 2: Suitability Summary

Vignette Response Summary Table: Suitability for iCare Home Monitoring

No. Responses n (%)				
**Vignette**	**Suitable for iCare Home**	**Unsuitable for iCare Home**	**No response regarding iCare Home**	**Total No. Responses**
**1**	26 (53)	23 (47)	0 (0)	49 (100)
**2**	28 (57)	18 (37)	3 (6)	
**3**	25 (51)	18 (37)	6 (12)	
**4**	30 (61)	14 (29)	5 (10)	

Vignette Response Summary Table: Suitability for OKKO Visual Field-Testing App

No. Responses n (%)				
**Vignette**	**Suitable for OKKO app**	**Unsuitable for OKKO app**	**No response regarding OKKO app**	**Total No. Responses**
**1**	28 (57)	19 (39)	2 (4)	49 (100)
**2**	26 (53)	18 (37)	5 (10)	
**3**	20 (41)	24 (49)	5 (10)	
**4**	32 (65)	12 (25)	5 (10)	

Vignette 5 Response Summary Table: Clinical Model Suitability

No. Responses n (%)				
**Vignette**	**Suitable Model**	**Unsuitable Model**	**No response**	**Total No. Responses**
**5**	26 (53)	18 (37)	5 (10)	49 (100)

5. Supplementary Table 3: Frequency and Duration Summary

Participant Response Summary Table for Frequency and Duration of iCare IOP Monitoring in Each Scenario

Scenario	Frequency of Monitoring	Frequency Comments (n)	Duration of Monitoring	Duration Comments (n)
**1**	**1-7 days**	**19**	1 month	2
	Monthly	3	**3-6 months**	**13**
	2-4 monthly	3	1 Year	4
	Other	1	Unclear	7
**2**	1-7 days	9	6 months	4
	1-3 monthly	5	1 Year	7
	**3-6 monthly**	**11**	**>1 Year**	**10**
	Yearly	1	Unclear	4
	Unclear	3	Other	4
**3**	**1-7 days**	**11**	1-2 weeks	5
	Monthly	3	**2-4 months**	**7**
	2-4 months	8	6 months	5
	Unclear	3	1 year	5
	Other	1	Unclear	2
**4**	Weekly	7	1-2 weeks	4
	Monthly	4	3-6 months	4
	**2-4 months**	**12**	**1 year**	**13**
	6-12 months	5	>1 year	4
	Unclear	2	Unclear	3

Table: 'Unclear' refers to comments which did not mention an explicit timeframe, 'Other' refers to timeframes which did not group with the remaining responses. Within each scenario, the timeframe with the most comments have been highlighted in bold.

<u>**Participant Response Summary Table for Frequency and Duration of OKKO VF app Monitoring in Each Scenario**</u>

Scenario	Frequency of Monitoring	Frequency Comments (n)	Duration of Monitoring	Duration Comments (n)
**1**	Weekly	5	<7 days	5
	**Monthly**	**9**	**2-4 weeks**	**8**
	2-4 monthly	8	2-4 months	9
	6 monthly	1	Yearly	5
	Unclear	5	Unclear	3
	Other	1	Other	3
**2**	1-3 months	8	1-2 weeks	2
	**6 Months**	**9**	6 months	3
	6-12 months	6	**12 months**	**8**
	Unclear	3	>12 months	6
	Other	2	Unclear	6
**3**	Weekly	2	Weekly	1
	Monthly	6	**3-6 months**	**11**
	**2-4 months**	**8**	1 Year	2
			>1 year	1
	Unclear	3	Unclear	4
**4**	Weekly	2	6 months	3
	Monthly	7	**1 year**	**16**
	2-4 months	8	>1 year	3
	**6-12 months**	**10**	Unclear	4
	Unclear	3	Other	2

Table: 'Unclear' refers to comments which did not mention an explicit timeframe, 'Other' refers to timeframes which did not group with the remaining responses. Within each scenario, the timeframe with the most comments have been highlighted in bold
