# Supplementary material for: Which glaucoma patients should be monitored at home and exploration of clinician perceptions on home monitoring? a survey of glaucoma specialists in the UK
Source: BMJ Open. 2024 Nov 18;14(11):e080873. doi: 10.1136/bmjopen-2023-080873 (PMC11574396; doi:10.1136/bmjopen-2023-080873)
Supplement: Supplementary file 3 [file bmjopen-2023-080873.reviewer_comments.pdf]

## PEER REVIEW HISTORY

BMJ Open publishes all reviews undertaken for accepted manuscripts. Reviewers are asked to complete a checklist review form and are provided with free text boxes to elaborate on their assessment. These free text comments are reproduced below.

### ARTICLE DETAILS

**Title (Provisional)**

Which Glaucoma Patients Should Be Monitored at Home and Exploration of Clinician Perceptions on Home Monitoring: A Survey of Glaucoma Specialists in the UK.

**Authors**

Alagappan, Uma; Stewart, Carrie; Azuara-Blanco, Augusto; King, Anthony J; Tatham, Andrew; Hernández, Rodolfo; MacLennan, Graeme; Shotton, Darian; Forrest, Mark; Gillies, Katie

---

### VERSION 1 - REVIEW

---

| | |
|---|---|
| **Reviewer** | **1** |
| **Name** | **Ho, Kam Chun** |
| **Affiliation** | **University of Canberra, Discipline of Optometry and Vision Science** |
| **Date** | **27-Nov-2023** |
| **COI** | **None** |

---

This study attempted to use clinical vignettes to identify target patients for home monitoring of glaucoma and whether it is feasible and accepted to “a range of stakeholders”. The authors could not draw a conclusion on whom would benefit from home monitoring of glaucoma with the four case scenarios. They also identify some facilitators and barriers using another case scenario. The current method that using case scenarios to answer the research question about feasible and acceptable of using home monitor technology for glaucoma does not seem to be the best method.

In the introduction, the authors should mention what types of monitoring are usually performed in glaucoma patients. Are there any studies showing the reliability of using iCare HOME and OKKO Health App? It is unclear why these two technologies were chosen or are these devices developed by the research team, so they would like to see the acceptance and feasibility of using such technologies for home monitoring? With the first aim being “identify suitable patients for glaucoma home monitoring”, the author should also introduce how important it is for patient selection and provide some references to support that. The author should also talk about the association between recommendations of home monitor

technologies and perception of clinicians on these technologies to support why they would like to survey the clinicians in this study. At the moment, the introduction does not strongly connect to the aims.

In the Method section, While the aim of the study is “to identify whether the home-monitoring of glaucoma is feasible and acceptable to a range of stakeholders through a mixed method feasibility study.”, only ophthalmologists and optometrists were invited instead of all stakeholders which should include patients. The authors should address why it is the case. For readers who are not familiar with the UK environment, can the authors describe a bit on how is the representative of members of UKEGS? Who are eligible to be a member of UKEGS? How many optometrists and ophthalmologists in UK? Why is it meaningful to survey them instead of all optometrists and ophthalmologists?

If the “clinical PIs raised awareness of the questionnaire amongst their own clinical networks and the study was promoted via social media”, how should the authors “accepting our denominator for calculating response rate as n=72.”? Based on that, the respondents are not necessarily a member of UKEGS especially there seemed no question in the questionnaire asking about whether the participants are members of the UKEGS.

“…and an introduction to the technologies being discussed (iCare Home Tonometer and the OKKO tablet-based App for measuring visual function) were included..” What types of information is provided? Are they peer-reviewed evidence? Are they just commercial leaflets introducing the products? All these can affect the responses of the participants, so the authors should provide more information on this.

Since the home monitoring technologies were not described in the NICE guidelines, it is unclear why the authors decided to use the clinical vignettes to investigate feasibility and acceptance of using such technology. As an exploring study, more open ended type of questions would gather more useful information than limited to 4 case scenarios. It is unclear the rationale why the authors decided to use such methodology, so the authors should provide some information on such decision and how valid the answers would be in answering the research question. Another issue is that the reasoning that a clinician decides to recommend home monitoring technology may not be based on the risk of the disease progression, but other aspects. The current study design would bias the responses and obtain very limited information.

In the Result section, it was reported that “Three participants were excluded based on not meeting our inclusion criteria of being involved in the treatment of glaucoma…” It further reinforced the problem of why the members of UKEGS were selected as some of the members were even not involved in glaucoma treatment although those three could be recruited via social media. The representative of the data was largely reduced and there seem no way to distinguish whether the responses were come from the UKEGS members or via other recruitment process.

| | |
|---|---|
| **Reviewer** | **2** |
| **Name** | **Guetterman, Timothy** |
| **Affiliation** | **University of Michigan Health System, Department of Family Medicine** |
| **Date** | **15-Dec-2023** |
| **COI** | **No known competing interests** |

Thanks for the opportunity to review this manuscript. The results of the study seem informative. I have suggestions regarding the methods and results presentations to strengthen the manuscript. They are detailed below.

Introduction:

The introduction includes a nice summary of home monitoring evidence and notes limitations regarding application to real world setting and who would benefit. However, it could more directly establish the need for this feasibility study. Moreover, explain why clinician perspectives are needed?

It is unclear whether the first aim referenced (p. 6) is the entire focus of the I-TRAC study or a portion of it. What is the relationship between this manuscript (I assume aim 1) and the larger study?

Methods:

Although the study is labeled mixed methods in its title, the methods do not reflect mixed methods. There is no mention of mixed methods design nor integration of the two. As written, it seems a multi-method survey.

Table 1 was helpful in understanding the vignettes and their distinction.

Why was 60% selected as the agreement criterion? Any evidence, literature, or background to support the selection would be helpful.

Please describe what is meant by text being "double reviewed". Did two individuals code the text responses?

Please review and revise the language around thematic analysis. You identified following thematic analysis and cited Braun and Clarke but reference "emergent themes", which does reflect the active process of the analyst. In addition, more information is needed to understand how you went from codes to themes.

Results:

Declaring "very limited agreement between clinicians" seems a bit too strongly worded. The percentage difference between scenarios seems relatively narrow at less than 10% between the scenario with the most and least Yes responses. Considering nonresponse and

measurement error, the percent Yes does not seem drastically different across scenarios in my opinion. Also, agreement might suggest agreement across scenarios (eg., if a respondent rated 1 a yes and 2 a no, others would tend to agree). Is agreement the right word?

I am confused by the results presented on p. 14 discussing the rationale for Scenario 4. Based on the description of participants, it seems like the same person had contradictory quotes. Please review and edit if needed. Otherwise, I might suggest finding quotes from other participants to show more breadth. Moreover, one of the quotes also appears in the table, which would be an opportunity to add a different illustrative quote.

Several themes need further development and more descriptive names. For example, “resources”, “patient characteristics”, “other” could be much more descriptive by detailing what you learned about the theme.

How does scenario 5 fit into results? It is mentioned on p. 17 in a paragraph before going into themes. However, are themes only based on Scenario 5?

There seems a disconnect between the themes and the table of barriers and facilitators. The text mentions that barriers and facilitators were identified for each theme, yet these are not labeled in the table. The footnote indicates B for barrier and F for facilitator, yet I do not see them in the table. Please describe how barriers and facilitators were identified (eg, in the methods describing analysis).

The results do not contain any mixed methods results. There may be opportunities to integrate, such as comparing the descriptives with themes.

Minor issues:

Define NHS at first use for the reader.

Define NIHR for the reader.

P. 4, ln 41, what is “this model”. Unclear what model you are referring to.

In Table 4, the first quote does not include racial/ethnic background.

P. 18, Table 2 –should this be Table 5?

---

## VERSION 1 - AUTHOR RESPONSE

| N | Reviewer 1 | Revision |
|---|---|---|
| 6 | This study attempted to use clinical vignettes to identify target patients for home monitoring of glaucoma and whether it is feasible and accepted to “a range of | No response required |

| | | |
|---|---|---|
| | stakeholders". The authors could not draw a conclusion on whom would benefit from home monitoring of glaucoma with the four case scenarios. They also identify some facilitators and barriers using another case scenario. | |
| **7** | The current method that using case scenarios to answer the research question about feasible and acceptable of using home monitor technology for glaucoma does not seem to be the best method. | As a stand-alone piece of research, we agree that the use of a survey using case scenarios would not be the 'best' method to answer the question about feasibility and acceptability of home monitoring. We have edited the manuscript to ensure that the aim of the research presented in this manuscript is clearly not presented as feasibility and acceptability work but rather a survey to investigate agreement amongst ophthalmologists about which glaucoma patients could be considered for home monitoring using digital technologies. We have chosen to retain the text describing the ITRAC study, which did assess acceptability and feasibility, to be able to provide the broader context in which this research sits.<br><br>See changes throughout.<br><br>The Title has also been updated to: ***Which Glaucoma Patients Should Be Monitored at Home: A Survey of Glaucoma Specialists in the UK*** |
| **8** | In the introduction, the authors should mention what types of monitoring are usually performed in glaucoma patients. | In current clinical practice, there are two main measurements used in the assessment of glaucoma are intraocular pressure measurement and visual field testing. Patients typically require lifelong monitoring and as per clinical recommendations are usually requested to attend monitoring every 6 months.<br><br>**This text has now been added to the introduction section – see page 4 lines 6-8.** |
| **9** | Are there any studies showing the reliability of using iCare HOME and OKKO Health App? It is unclear why these two technologies were chosen or are these devices developed by the research team, so they would like to see the acceptance and feasibility of using such technologies for home monitoring? | **We have included text within the methods to describe the rationale for the use of the two digital technologies. See page 7 line 9-20:**<br><br>The two technologies that were initially selected to be explored within this study were the iCare HOME tonometer, to measure IOP, and the MRF app, accessed via an iPad to measure visual fields. However, the MRF app was not CE marked so instead replaced with the OKKO health app. |

| | | |
|---|---|---|
| | | We predicted that the OKKO health app would have transferrable findings to the original MRF app, considering it is also an app-based visual fields device, but however understand that the lack of assessment was not ideal. We also note that the lack of published evidence about the OKKO app is a limitation.<br><br>The iCare home tonometer has been studied to suggest that most participants were able to correctly use the device following training. Additionally, the iCare HOME tonometer has been compared against the Goldmann automated tonometer (GAT) in numerous studies. Overall, the measurement differences reported between GAT and iCare vary between -2.7 to 0.7mmHg. Importantly, variations of 0-5mmHg are considered acceptable ranges for home monitoring. |
| **10** | With the first aim being "identify suitable patients for glaucoma home monitoring", the author should also introduce how important it is for patient selection and provide some references to support that. | **We have now included text to highlight this point. See page 4 line 28-33:**<br><br>Evidence suggests that the demand for glaucoma services will continue to expand, with longer monitoring periods predicted in the future. This has led to delays in follow-up appointments, ultimately resulting in evidence showing irreversible visual loss which could have been prevented with adequate monitoring. There is limited guidance in the literature as to which patients would be the ideal for home-monitoring. Identifying uncertainties regarding patient suitability is a critical first step towards evaluating its use. |
| **11** | The author should also talk about the association between recommendations of home monitor technologies and perception of clinicians on these technologies to support why they would like to survey the clinicians in this study. | **We have now included text in the introduction to highlight this point. See page 4 lines 16-18, 31-33:**<br><br>Several qualitative studies have reported successes for chronic conditions such as diabetes and hypertension.<br><br>There is limited guidance in the literature as to which glaucoma patients would be the ideal candidates for home monitoring using digital technology. Identifying key uncertainties regarding patient suitability is a critical first step towards evaluating its use. |
| **12** | At the moment, the introduction does not strongly connect to the aims. | We believe that given the changes requested above by the reviewer that the introduction now more closely reflects the revised aims to determine whether there is agreement amongst clinicians regarding which glaucoma patients may be suitable for home monitoring and assessing clinicians overall perspectives of home monitoring in glaucoma care. |

| | | |
|---|---|---|
| **13** | In the Method section, While the aim of the study is "to identify whether the home-monitoring of glaucoma is feasible and acceptable to a range of stakeholders through a mixed method feasibility study.", only ophthalmologists and optometrists were invited instead of all stakeholders which should include patients. The authors should address why it is the case. | This comment has been addressed through an edit to the aims to make it clear that the survey study reported in this manuscript was concerned with identification of suitable patients rather than acceptability and feasibility of the digital **technologies more broadly (an aim of the parent study, ITRAC).**<br><br>We have included text to indicate why optometrists and ophthalmologists were invited to participate in the survey. **Please see page 6 lines 7-12.**<br><br>As such, ophthalmologists and optometrists were invited to participate within the survey as they are directly involved in the monitoring and management of glaucoma patients, therefore allowing clinically relevant opinions to be evidenced.<br><br>We agree that patients are key stakeholders within this research topic. As a part of the larger ITRAC project, patients were invited to participate in interviews to offer their opinion on the technologies and additionally trialled the technologies' themselves for a period of 3-months. Following this, they were invited to attend further discussions regarding their perspective after using the devices for 3-months. These results are explored within the ITRAC monograph. |
| **14** | For readers who are not familiar with the UK environment, can the authors describe a bit on how is the representative of members of UKEGS? Who are eligible to be a member of UKEGS? How many optometrists and ophthalmologists in UK? Why is it meaningful to survey them instead of all optometrists and ophthalmologists?<br><br>If the "clinical PIs raised awareness of the questionnaire amongst their own clinical networks and the study was promoted via social media", how should the authors "accepting our denominator for calculating response rate as n=72."? Based on that, the respondents are not necessarily a member of UKEGS especially there seemed no question in the questionnaire asking about whether the participants are members of the UKEGS. | **We have included text to add some background on UKEGS and the response rate. See page 6 lines 16-26.**<br><br>Based upon the estimated number of glaucoma clinicians registered with UK and Eire Glaucoma Society (UKEGS), a non-profit professional society for clinicians with a specialist interest in glaucoma (range n=69-72) we are accepting our denominator for calculating ESTIMATED response rate as n=72. UKEGS does not currently record the designation of its members, so the exact number of glaucoma consultants surveyed is unknown.<br><br>In order to target clinicians with a focussed interest in glaucoma research, the survey was disseminated via UKEGS. A link to the questionnaire with an invitation to participate was emailed to members of UKEGS by the UKEGS Communications Manager. In addition to the invitation email, the clinical co-investigators raised awareness of the questionnaire amongst existing clinical networks. The survey was active from 14$^{th}$ May 2021 to 30$^{th}$ October 2021. |

| | | |
|---|---|---|
| **15** | "…and an introduction to the technologies being discussed (iCare Home Tonometer and the OKKO tablet-based App for measuring visual function) were included.." What types of information is provided? Are they peer-reviewed evidence? Are they just commercial leaflets introducing the products? All these can affect the responses of the participants, so the authors should provide more information on this. | Information on ITRAC and home-monitoring devices was included at the start of the survey to ensure participants were given contextual insights to promote informed responses. Additionally, a summary of the National Institute for Health and Care Excellence (NICE) guidelines for ocular hypertension (OHT) and primary open angle glaucoma (POAG) and an introduction to the technologies being discussed (iCare Home Tonometer and the OKKO tablet-based App for measuring VFs) were included. Peer reviewed evidence for the iCare tonometer was referenced within the leaflet but there is yet to be published evidence regarding the OKKO app. (please see comment 9).<br><br>**This has been included in the methods section on page 7, lines 23-29.** |
| **16** | Since the home monitoring technologies were not described in the NICE guidelines, it is unclear why the authors decided to use the clinical vignettes to investigate feasibility and acceptance of using such technology. As an exploring study, more open ended type of questions would gather more useful information than limited to 4 case scenarios. It is unclear the rationale why the authors decided to use such methodology, so the authors should provide some information on such decision and how valid the answers would be in answering the research question. | The additional changes made to the framing of the aim of this survey will now address some aspects of this comment i.e. not to describe study as acceptability and feasibility but place it in wider context of ITRAC.<br><br>To further justify the choice of study design for our survey, we utilised a mixture of both open and close-ended questions within our four clinical vignettes to try to obtain a wide variety of responses regarding four differing but likely patient scenarios. If the scenarios were left completely open ended, the responses may have been too wide to collate any valuable themes for analysis. Scenario 5 was proposed in aim of identifying perceived barriers and facilitators of home monitoring (allowing creation of themes through the free text responses), whereas the clinical vignettes were utilised in attempt of researching whether there was agreement amongst clinicians regarding which type of patients could be suitable for home monitoring.<br><br>**Text has been added to the methods section to further explain this, please see Page 6 Line 30-33, page 7 lines 1-4, page 9 lines 4-8.** |
| **17** | Another issue is that the reasoning that a clinician decides to recommend home monitoring technology may not be based on the risk of the disease progression, but other aspects. The current study design | We recognise this limitation but also hope that the editing of the aim of this survey again helps to address this point. We have included in the discussion that the larger ITRAC study identified other aspects which may determine patient selection for home monitoring |

| | | |
|---|---|---|
| | would bias the responses and obtain very limited information. | which will be published within the monograph. **Please see response to comment 13** |
| **18** | In the Result section, it was reported that "Three participants were excluded based on not<br>meeting our inclusion criteria of being involved in the treatment of glaucoma…" It further reinforced the problem of why the members of UKEGS were selected as some of the members were even not involved in glaucoma treatment although those three could be recruited via social media. The representative of the data was largely reduced and there seem no way to distinguish whether the responses were come from the UKEGS members or via other recruitment process. | We recognise that the survey did not include a question at the start to determine where the respondent heard about the survey (e.g. through a direct source or UKEGS). However, the survey did include initial screening question to ensure that only respondents directly involved in glaucoma care could proceed forward in the survey. Following this, further questions were included to determine occupation etc, ensuring only relevant respondents were selected for analysis.<br><br>**Above text has been explained in Page 19, lines 26-30.**<br><br>**The below text has also been added to justify exclusion of other participants based on not meeting our inclusion criteria one Page 11 Line 3-6:**<br><br>The three participants who were excluded based on lack of meeting inclusion criteria were excluded from analysis due to factors such as their professional role. For example, glaucoma nurses were excluded as they did not directly make clinical decisions regarding monitoring or management for glaucoma patients, despite having an interest in glaucoma care and working with patients regularly. |

| N | Reviewer 2 | Revision |
|---|---|---|
| **19** | Thanks for the opportunity to review this manuscript. The results of the study seem informative. I have suggestions regarding the methods and results presentations to strengthen the manuscript. They are detailed below. | No response required |
| **20** | Intro could more directly establish the need for this feasibility study. Moreover, explain why clinician perspectives are needed? | Given the edits in response to reviewer 1 above in relation to the aim being disconnected form the introduction, we believe many of those associated changes also address this comment. In addition, we have included the following text in the introduction on **page 4, Lines 23-26** regarding clinicians' |

| | | |
|---|---|---|
| | | perspectives, **please see comment 11.** |
| **21** | It is unclear whether the first aim referenced (p. 6) is the entire focus of the I-TRAC study or a portion of it. What is the relationship between this manuscript (I assume aim 1) and the larger study? | The first aim of the I-TRAC study, explored within this manuscript, was to identify which patient would be suitable for glaucoma home monitoring through a survey to investigate agreement amongst clinicians and to explore their perceptions of the possible benefits and risks of home monitoring devices. Please see responses to reviewer 1 comments above (comment 13) that also relates to a lack of clarity on this issue.<br><br>**Please see introduction Page 5 Lines 1-10 for further clarity.** |
| **22** | Although the study is labelled mixed methods in its title, the methods do not reflect mixed methods. There is no mention of mixed methods design nor integration of the two. As written, it seems a multi-method survey. | **We retain that this study utilises a mixed-methods design and have included the below text on page 6-7 lines 30-33 and 1-4, respectively, to justify this:**<br><br>The online questionnaire utilised a mixed-methods design. The initial data collection used a combination of closed-ended vignettes, to quantitatively investigate which patient's clinicians would deem suitable for home monitoring, and open-ended free-text questions to assess overall perspectives. Following this, separated data analysis permitted generation of quantitative frequencies and percentages in relation to agreement amongst clinicians regarding patient selection whilst also qualitatively assessing free-text responses regarding home-monitoring for theme creation. Findings were then mixed at the interpretation phase by justifying quantitative findings with qualitative responses. |

| | | |
|---|---|---|
| | | Please additionally see this figure below for further clarity.<br>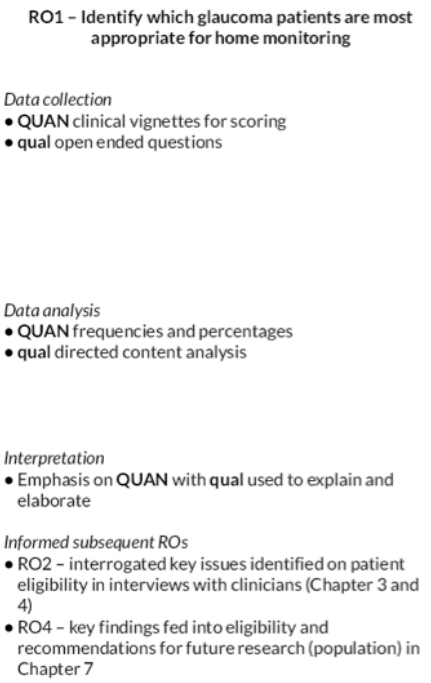<br> |
| **23** | Why was 60% selected as the agreement criterion? Any evidence, literature, or background to support the selection would be helpful. | **The following text has been included in the methods section, see page 9 lines 29-32.**<br><br>Quantitative data was analysed using descriptive statistics (e.g. frequencies, percentages). Agreement within clinical scenarios was defined by the study team as being ≥ 60% in supporting or not supporting the hypothetical patient to be home-monitored. We chose this value based on team discussion and on best judgement that over half of the respondents agreed. |
| **24** | Please describe what is meant by text being “double reviewed”. Did two individuals code the text responses? | **Text to make this explicit has been added to page 10 lines 11-14 as below:**<br><br>UA and CS reviewed the open response data, noting the points being made in each response as codes, which were then reviewed for similarities and differences. UA and CS then developed a list of themes, groups of interconnected |

| | | |
|---|---|---|
| | | codes, for both barriers and facilitators. Once provisional themes were developed UA wrote up a coding framework to define each theme. |
| **25** | Please review and revise the language around thematic analysis. You identified following thematic analysis and cited Braun and Clarke but reference "emergent themes", which does reflect the active process of the analyst. In addition, more information is needed to understand how you went from codes to themes. | **The below text has been summarised within methods to further expand on this comment. Please see Page 10 Lines 1-23:**<br><br>Thematic content analysis was used to qualitatively analyse the content of open response content. The 6-Phase Braun and Clarke approach to thematic analysis was adopted. We used both inductive and deductive coding to build themes, useful units of data to explain the findings.  For inductive, codes are developed based upon searching for similar issues or points within the data. For deductive codes we looked for existing concepts and ideas based upon previous work, in this case, limited information in relation to clinicians' attitudes towards and use of technologies for home monitoring. For both inductive and deductive coding, we were review the data from the perspective of identifying barriers and facilitators. Barriers were defined as features of the intervention itself or the environment it would be implemented within which can or has potential to prevent or limit the utility of the intervention. Facilitators were defined as features of the intervention itself or the environment it would be implemented within which can or has potential to permit or enhance the utility of the intervention.<br><br>UA and CS reviewed the open response data, noting the points being made in each response as codes, which were then reviewed for similarities and differences. UA |

| | | |
|---|---|---|
| | | and CS then developed a list of themes, groups of interconnected codes, for both barriers and facilitators. Once provisional themes were developed UA wrote up a coding framework to clearly define each theme. Themes are generally descriptive rather than analytical, reflecting the limited qualitative data available and the inability to check understanding or explore points raised further. This was intentional so as to link the findings from this work to subsequent phases of ITRAC and to use these descriptive themes as areas for further exploration in the interviews and focus groups. We don't believe that the data from the free text responses in the survey were rich enough for the development of in-depth conceptual themes. Descriptive themes often incorporate both barriers and facilitators (as illustrated in the framework) reflecting divergence in views of participants.<br><br>The development of themes was supported by use of QSR NVivo programme. The coding framework, listing the themes their codes and their descriptions, was updated throughout the analytical process. For rigour, themes were reviewed and agreed by the team. |
| **26** | Declaring "very limited agreement between clinicians" seems a bit too strongly worded. The percentage difference between scenarios seems relatively narrow at less than 10% between the scenario with the most and least Yes responses. Considering nonresponse and measurement error, the percent Yes does not seem drastically different across scenarios in my opinion. | We have reworded from "very limited agreement between clinicians" to "Agreement amongst clinicians could not be determined" to clarify our study findings. **See page 12 lines 2-3.** |
| **27** | Also, agreement might suggest agreement across scenarios (eg., if a respondent rated 1 | We have chosen to retain the word agreement as the aim of the study |

| | | |
|---|---|---|
| | a yes and 2 a no, others would tend to agree). Is agreement the right word? | was to determine agreement amongst clinicians regarding which patient population would be best suited to home monitoring. However, we have specified that this relates to agreement within scenarios rather than across. **See page 12 lines 2-3.** |
| **28** | I am confused by the results presented on p. 14 discussing the rationale for Scenario 4. Based on the description of participants, it seems like the same person had contradictory quotes. Please review and edit if needed. Otherwise, I might suggest finding quotes from other participants to show more breadth. Moreover, one of the quotes also appears in the table, which would be an opportunity to add a different illustrative quote. | In relation to the results on page 14, two separate respondents (with same demographics) gave differing opinions regarding rationale for scenario 4 that were both used for analysis. These may appear to be contradicting quotes from the same respondent but, to clarify, they are instead two separate responses from two separate respondents. Please also not that respondent demographics have been edited to now only reflect professional role and years of experience, **as per comment 4.**<br><br>**The repeated quote has now been removed from Table 4 on page 15 and replaced with the below quote:**<br><br>*'Low risk - not worth the extra resources'* – consultant with >10 years' experience |
| **29** | Several themes need further development and more descriptive names. For example, "resources", "patient characteristics", "other" could be much more descriptive by detailing what you learned about the theme. | The theme generation in this project was descriptive (**and has now been clarified in methods page 10, lines 14-19**). This was intentional so as to link the findings from this work to subsequent phases of ITRAC and to use these descriptive themes as areas for further exploration in the interviews and focus groups. We don't believe that the data from the free text responses in the survey were rich enough for the development of in-depth conceptual themes. |
| **30** | How does scenario 5 fit into results? It is mentioned on p. 17 in a paragraph before | Scenario 5 was designed in order to gather responses on overall |

| | | |
|---|---|---|
| | going into themes. However, are themes only based on Scenario 5? | opinions of glaucoma home monitoring, that were then analysed to create the themes introduced on **pages 16-18**. The individual vignettes were designed to assess whether clinicians had any agreement on which types of patients may be suitable for home monitoring but did not contribute towards themes. **This has been clarified on Page 9 Lines 4-9.** |
| **31** | There seems a disconnect between the themes and the table of barriers and facilitators. The text mentions that barriers and facilitators were identified for each theme, yet these are not labeled in the table. The footnote indicates B for barrier and F for facilitator, yet I do not see them in the table.<br><br>Please describe how barriers and facilitators were identified (eg, in the methods describing analysis). | We now have edited the table to include B for barrier and F for facilitator as per the footnote. Please see **Page 17-18.**<br><br>**We have included updated text on Page 10 Lines 4-9 to define barriers and facilitators within the context of this manuscript.**<br><br>Barriers are features of the intervention itself or the environment it would be implemented within which can or has potential to prevent or limit the utility of the intervention. Facilitators are features of the intervention itself or the environment it would be implemented within which can or has potential to permit or enhance the utility of the intervention. |
| **32** | The results do not contain any mixed methods results. There may be opportunities to integrate, such as comparing the descriptive with themes. | Please see response to **comment 22** |
| **33** | Define NHS at first use for the reader.<br><br>Define NIHR for the reader.<br><br>P. 4, ln 41, what is "this model". Unclear what model you are referring to.<br><br>In Table 4, the first quote does not include racial/ethnic background.<br><br>P. 18, Table 2 –should this be Table 5? | The points below have been edited within the manuscript.<br><br>National health service - **page 4, line 9**<br><br>National institute of health research – **Page 5, lines 1-2** |

| | | |
|---|---|---|
| | | This model refers to home monitoring as a model of care– **edited on page 4, lines 20-21**<br><br>Quote 1 table 4: will be limiting quotes to only professional role and years of experience, as discussed in **comment 4.**<br><br>We have reviewed **page 18** and ensured both the title and legend are labelled Table 5. |

## VERSION 2 - REVIEW

**Reviewer** 2

**Name** Guetterman, Timothy

**Affiliation** University of Michigan Health System, Department of Family Medicine

**Date** 15-Apr-2024

**COI** No known competing interest.

I found this paper on Glaucoma specialists' views of home monitoring to be interesting and potentially a valuable contribution the the larger body of literature on remote monitoring and telehealth. It could fill an important gap. I did notice weaknesses related to the mixed methods design, description of anlaysis, and reporting of results.

-The paper proposes to use a mixed methods design, but goes into little detail. It seems to be a convergent design, though not specified, given the survey. Despite proposing to be a mixed methods study, it lacks a description of integration. The methods should identify mixed methods integrative anlaysis strategies, and the results should report the mixed methods results.

-The stated objective varies throughout. The abstract objective focuses on clinicians' views of home monitoring, which seems accurate. However, the paper mentions an objective to investigate agreement. The former seems more in line what was done. If the goal was to assess agreement, it is unclear how that was assessed in addition to any compensation for chance agreement. Moreover, the results do not report specific agreement measures, but only a broad summary of whether the 60% threshold was met. Personally, I would find a paper on views more interesting than one focused on agreement only.

-The results would benefit from re-organization. It was unclear what themes were overall. They are reported under acceptability, yet quotes are also included elsewhere under the heading, "Can a target patient population for glaucoma home-monitoring be established?" What themes did these quotes related to? Moreover, the themes identified under acceptability and in the table are not descriptive. Given the methods following Braun and Clarke's thematic analysis, it seems more attention naming themes would help.

-As noted above, the integrated mixed methods results are not clear nor discussed fully. What was learned from integrating?

-The paper contains two tables labeled Table 2.

---

## VERSION 2 - AUTHOR RESPONSE

| N | Peer Reviewer Comment | Response |
|---|---|---|
| 1 | I found this paper on Glaucoma specialists' views of home monitoring to be interesting and potentially a valuable contribution the larger body of literature on remote monitoring and telehealth. It could fill an important gap. I did notice weaknesses related to the mixed methods design, description of analysis, and reporting of results. | No response required. |
| 2 | The paper proposes to use a mixed methods design but goes into little detail. It seems to be a convergent design, though not specified, given the survey. Despite proposing to be a mixed methods study, it lacks a description of integration. The methods should identify mixed methods integrative analysis strategies, and the results should report the mixed methods results. | We would like to clarify that our **larger parent study, I-TRAC,** is a multi-phase, **mixed-method study** of acceptability and feasibility of glaucoma home monitoring. We have clarified this within the introduction text on **Page 5, Lines 1-11.**<br><br>However, this manuscript pertains to **one component** of the larger ITRAC study, which was an online survey, comprising of both closed and open response questions. This study aimed to identify which patients' clinicians deemed suitable for glaucoma home monitoring and to explore clinicians' **perceptions** (see response to **comment 3**) of the possible benefits and risks of home monitoring devices. The responses to these questions were explored with quantitative methods, utilising descriptive statistics (e.g. frequencies and percentages), and thematic analysis of open-ended responses. We have clarified this in the introduction (page and line numbers as above) and methods - please see **Page 9, Line 29 to Page 10, Line 24**. We have also edited our results titles to |

| | | |
|---|---|---|
| | | maintain consistency (please see response to **comment 4**).<br><br>We have now removed mention of 'mixed methods' from throughout our text, in reference to this manuscript, and retained that the survey was comprised of open and closed ended responses that were analysed to give descriptive statistics and themes/codes to avoid any confusion. |
| **3** | The stated objective varies throughout. The abstract objective focuses on clinicians' views of home monitoring, which seems accurate. However, the paper mentions an objective to investigate agreement. The former seems more in line what was done.<br><br>If the goal was to assess agreement, it is unclear how that was assessed in addition to any compensation for chance agreement.<br><br>Moreover, the results do not report specific agreement measures, but only a broad summary of whether the 60% threshold was met. Personally, I would find a paper on views more interesting than one focused on agreement only. | We now have re-phrased our objectives and maintained this consistently throughout the text. The new objective is: *'to identify suitable patients for glaucoma home monitoring and explore clinicians' perceptions of the possible benefits and risks of home monitoring.'*<br><br>This has been retained throughout the text, particularly within the abstract (**Page 2, Line 3-5**), introduction (**Page 5, Lines 8-11**) and results (please see response to **comment 4**), to better encompass the findings of the manuscript and maintain consistency.<br><br>The 60% threshold was devised from clinician group consensus. We have now ensured to reframe our manuscript objectives to additionally explore clinicians' **perceptions** of glaucoma home monitoring, rather than solely agreement, to address this comment. Please see Page and Line numbers as above. |
| **4** | The results would benefit from re-organization. It was unclear what themes were overall. They are reported under acceptability, yet quotes are also included elsewhere under the heading, "Can a target patient population for glaucoma home-monitoring be established?" What themes did these quotes related to? | We have arranged our results based on our now edited objectives, as above in comment 3. Please see the updated results titles below:<br><br>1 'Can a target group of patients who would be most suited for glaucoma home monitoring can be defined', please see **Page 12, Line 1** – the quotes from this section are taken from **only from the four clinical vignettes** designed to investigate whether a target patient population could be determined for glaucoma home monitoring. The results presented in this section utilise both closed and open-ended responses.<br><br>2 'The acceptability of glaucoma home monitoring from the perspective of glaucoma specialist clinicians', please see **Page 16, Line 1** – the quotes and themes included in this |

| | | |
|---|---|---|
| | | section were gathered from **only the open-ended questions** regarding clinicians overall **general perceptions** of glaucoma home monitoring fitting into the current healthcare system and the associated benefits and risks of this. |
| **5** | Moreover, the themes identified under acceptability and in the table are not descriptive. Given the methods following Braun and Clarke's thematic analysis, it seems more attention naming themes would help. | We have now edited our theme names to be more descriptive. For example, the theme of 'Accessibility' has been updated to 'Clinician concerns regarding the impact of home monitoring for patients with accessibility barriers.'<br><br>Please see **Table 5 on Page 17.** |
| **6** | As noted above, the integrated mixed methods results are not clear nor discussed fully. What was learned from integrating? | Please see response to **Comment 2** to clarify this. |
| **7** | The paper contains two tables labelled Table 2. | We have amended this in our text. |

---

## VERSION 3 - AUTHOR RESPONSE

Good afternoon,

Thank you for your ongoing consideration of our manuscript.

We have received editorial comments on a previous revision to edit our heading of 'Table 2' to 'Table 5' on page 17. We have emailed the editorial team in response seeking support as our original submitted files contain the correct heading of 'Table 5' but the PDF proof converts this title to 'Table 2'. The editorial team believe this is due to a bug and have advised us to re-submit our manuscript with this point addressed in the covering letter in hope of amending this with the production team if the manuscript is accepted.

We have now updated our heading in the main text to 'Patient and Public Involvement Statement' and have formatted our author contributorship statement into a paragraph as per the last editorial comments.

Best wishes,

Dr. Uma Alagappan
